# Acyclic and cyclic imines and their metal complexes: recent progress in biomaterials and corrosion applications

**DOI:** 10.1039/c8ra01890a

**Published:** 2018-06-27

**Authors:** Wail Al Zoubi, Saad Gomaa Mohamed, Abbas Ali Salih Al-Hamdani, Agastya Prastita Mahendradhany, Young Gun Ko

**Affiliations:** Materials Electrochemistry Group, School of Materials Science and Engineering, Yeungnam University Gyeongsan 38541 Republic of Korea wailalzoubi@ynu.ac.kr younggun@ynu.ac.kr; Mining and Metallurgy Engineering Department, Tabbin Institute for Metallurgical Studies (TIMS) Tabbin Helwan 109 Cairo 11421 Egypt; Department of Chemistry, College of Science for Women University of Baghdad Baghdad Iraq

## Abstract

This review describes the contemporary development applications on scientific areas of acyclic and cyclic Schiff bases and their complexes with an emphasis on the author’s contribution to the field. After a short historical introduction, this manuscript is divided into two main parts. In the first section, Schiff bases are reviewed for their biological activities including antibacterial, antifungal, antioxidant, cytotoxic, and enzymatic activities as well as their interaction with single-stranded-DNA which have shown remarkable activities in each region of research. The second part deals with the corrosion of metal and its alloys in corrosive environments and their organic inhibitors. The main section of this part is to investigate the different chemical structures for Schiff bases used in such aggressive solution to protect metals. Knowing the maximum corrosion efficiency or bioactivity of a specific chemical structure in a specific application environment is helpful for choosing the most appropriate compound.

## Abstract 2

The contemporary development applications on scientific areas of acyclic and cyclic Schiff bases and their complexes.

## Graphical abstract

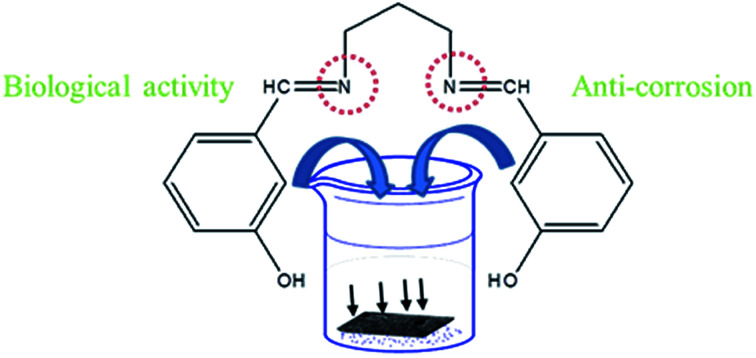

## 1. Introduction

The term Schiff base (SB) comes from the name of Hugo Schiff,^1,2^ German by nationality, who prepared the first so-called Schiff base in 1864.^3,4^ Schiff bases (SBs) contain the azomethine group (–RC=N–) and are typically synthesized by condensation reactions of an active carbonyl compound with a primary amine. They are considered to be a promising candidate for a variety of applications related to biological activities (such as antiapoptotic, antifungal, antibacterial, anti-inflammatory and antiviral activities),^5–7^ catalytic activities,^8–13^ electroluminescent properties,^14–16^ fluorescence properties,^17–19^ nonlinear optical (NLO) properties,^20^ and applications in electrochemical sensing^21^ and organic photovoltaic materials.^22^ Owing to the ease of preparation and tunability of their stereo-electronic structures, most SBs are attractive ligands because they readily form stable complexes with most metal ions.^23^ In addition, SB ligands are able to coordinate with various metal ions thus stabilizing them in different oxidation states, and, therefore they are among the most important ligands used in current coordination chemistry due to their well-known coordinative capability. Due to imine (CH=N), hydroxyl (OH) and other groups SBs possess an important position in the biological field.^24,25^ As a consequence, a broad research field dealing with cyclic and acyclic SBs and their metal complexes has led to an extensive number of works, and has been comprehensively studied in 2017^26^ and 1987.^27^ In 2004, Hernandez-Molina and co-workers^28^ also reported some classical synthetic methods of asymmetric SBs derived from diaminomaleonitrile and their Zn, Cd and Cu complexes. This provides a more general overview of the chemical synthesis of SBs, the preparation of various corresponding metal complexes and their different applications. The main goal of this work is to give a complementary yet more detailed overview on acyclic and cyclic SBs and their metal complexes, focusing strongly on compounds for their application as bioactive components and organic inhibitors. On the other hand, this review paper also focuses on organic inhibitors in corrosive environments and not inside the coatings. Therefore, we try to collect main experimental results of researches about metal corrosion inhibition in different Schiff bases, to present comprehensive insight for researchers with appropriate categories and to provide guidance for future studies in this field.

## 2. Biological and anti-corrosion applications of acyclic and cyclic Schiff bases

Azomethine or imine groups present in the organic compounds have been shown to be critical to their biological activities. In addition, the presence of nitrogen and oxygen donor atoms in such compounds makes them structurally similar to neutral biological systems and they are utilized in elucidating the mechanism of transformation of racemization reactions.^29–31^ Various biological activities are considered due to the presence of the azomethine linkage (>C=N–) present in living systems.^32^ On the other hand, for anti-corrosion, the corrosion inhibition properties of these compounds can be assigned to its molecules with π-electrons of the imine group and π-electrons of aromatic substituents.

### 2.1. Biological activities of acyclic and cyclic Schiff bases

Schiff bases and their complexes are considered as a special category of compounds owing to their biochemical synthesis, antimicrobial, antifungal, electrochemical analysis as well as catalytic activities.^33^ Therefore, various SBs forming metal complexes have been comprehensively studied, exhibiting a broad range of applications, especially in biological systems. The study of SBs and their complexes reveals their interesting spectral properties which are helpful in understanding the various features of the coordination chemistry of metal ions. On the other hand, previous works performed in the last decades have recognized the ability of SBs and their complexes to exert various bioactive effects either *in vitro* or *in vivo*. Details on these applications are given below.

#### 2.1.1 Schiff bases containing aromatic moieties

**Scheme 1:**
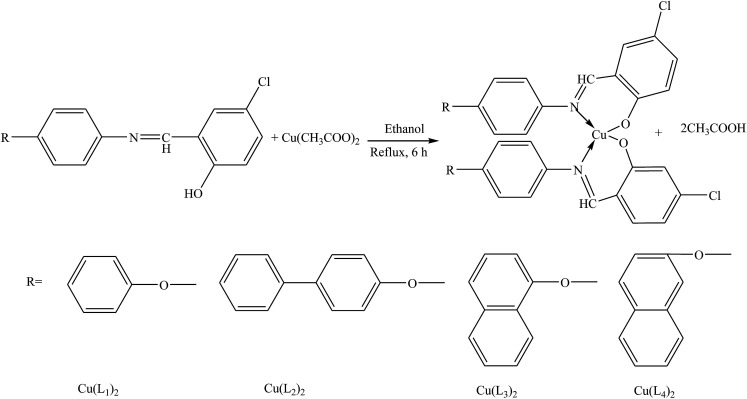
Synthesis of Cu(ii) complexes [Cu(L_1_)_2_–Cu(L_4_)_2_].

Shabbir *et al.* have reported the synthesis of four Schiff base Cu(ii) complexes, *i.e.* bis((*E*)-2-((4-phenoxyphenylimino)methyl)phenolate) copper(ii) (Cu(L_1_)_2_), bis((*E*)-2-((4-(4-biphenyloxy)phenylimino)methyl)phenolate) copper(ii) (Cu(L_2_)_2_), bis((*E*)-2-((4-(naphthalen-1-yloxy)phenylimino)methyl)phenolate) copper(ii) (Cu(L_3_)_2_) and bis((*E*)-2-((4-(2-naphthoxy)phenylimino)methyl)phenolate) copper(ii) (Cu(L_4_)_2_) (Scheme 1). Biological researches (cytotoxic and antitumor) have shown that these complexes are bioactive in these environments. Therefore, the SBs are highly active in protecting DNA against ˙OH radicals in a concentration-dependent way, *i.e.* DNA defense is increased with the increase in the concentration of the test compound. In addition, SBs exhibited significant activity against brine shrimp nauplii.^34,35^ Further, the prepared SBs and their complexes seem to be able to combine with the lipophilic layer in order to enhance the membrane permeability of biological applications.

**Scheme 2:**
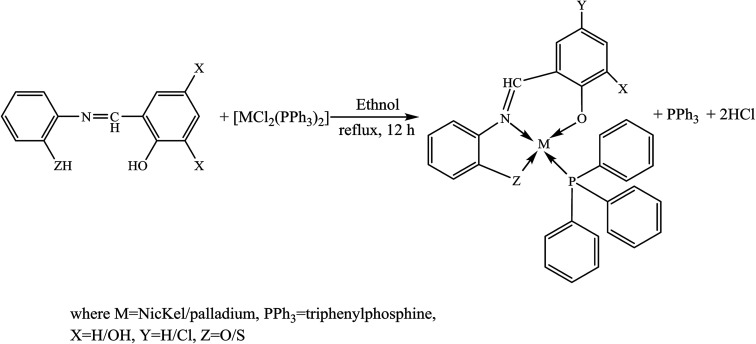
Synthesis of complexes M(L_1_)PPh_3_–M(L_4_)PPh_3_.

On the other hand, the incorporation of heavy metal ions such as Ni(ii) and Pd(ii) triphenylphosphine (Scheme 2) in tridentate SBs has been successfully carried out. The organometallic moieties clearly improved the biological performance, *e.g.*, in antimicrobial assays Ni(L_1_)PPh_3_ exhibited dual inhibition against bacterial and fungal strains whereas for the rest of the compounds varying the degree of activity was examined against different strains. The prepared compounds have shown a high tendency for DNA binding either through intercalation or a groove-binding nature which displays the mechanism of the antitumor effect of these metal complexes. These findings support the view that some of these compounds can be promising candidates for drug formulation and development due to their diversity of bioactivities.^36^

**Scheme 3:**
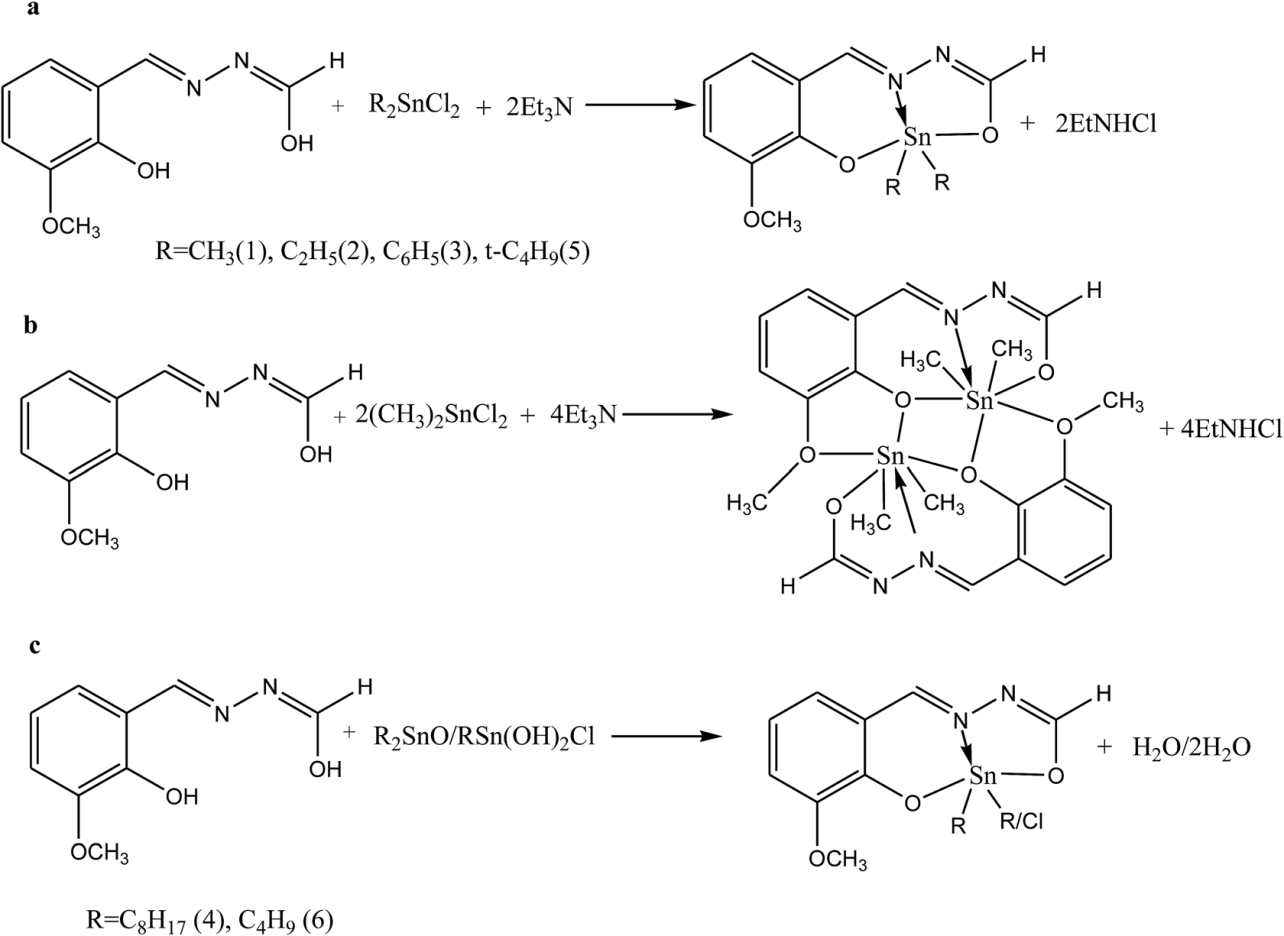
Synthesis of Schiff base complexes (a–c).

**Scheme 4:**
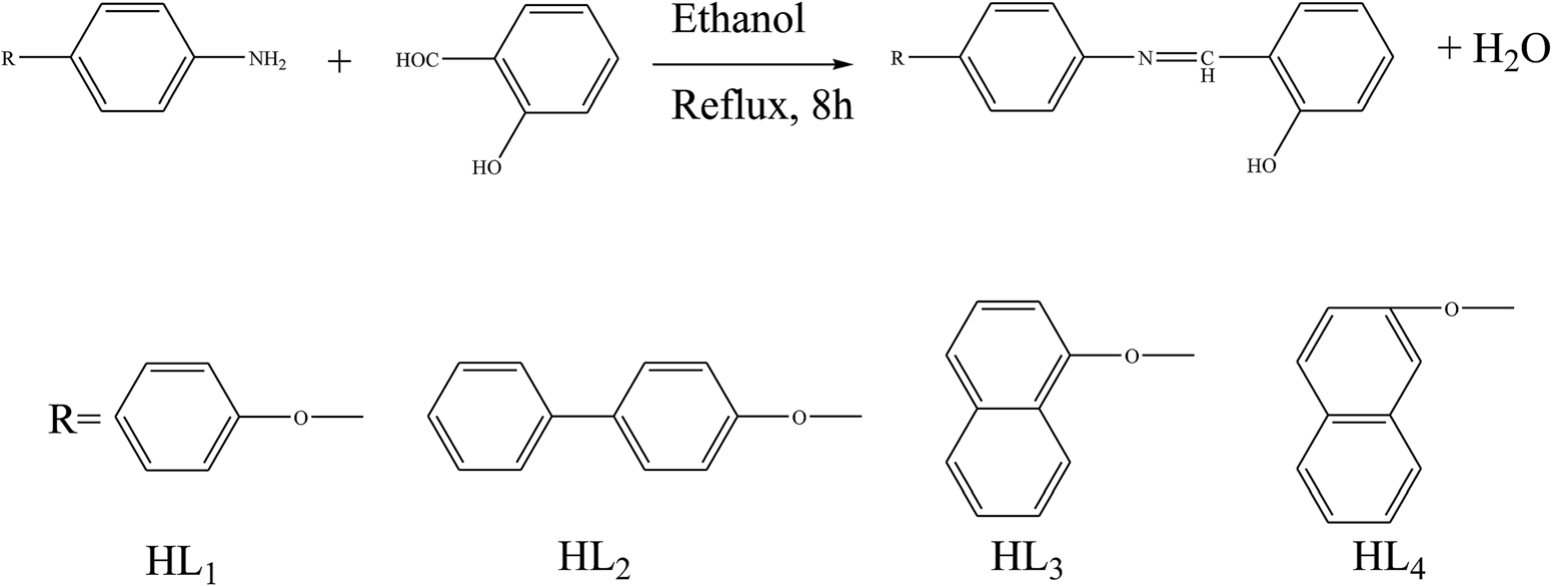
Synthesis of imines (HL_1_–HL_4_).

Shujah *et al.*^37^ have reported the synthesis and bioactivity of dimethyl (1), diethyl (2), diphenyl (3), di-*n*-octyl phthalate (4), di-*tert*-butyl (5), and *n*-butylchlorotin(iv) (6) derivatives of *N*′-(2-hydroxy-3-methoxybenzylidene) form hydrazide ligand (Scheme 3a–c). These compounds are either mononuclear in which the Sn atom is in a distorted trigonal bipyramidal geometry (a and c) or homobimetallic with each Sn atom in a pentagonal bipyramidal environment (b). This behavior can be assigned to the steric factor and the high lipophilic character in the former. These factors, planarity and low molecular weight, which facilitate smooth diffusion were presumably turned into the high activity of diphenyltin(iv) complexes (3). Besides, the chemical structure suggests that the H⋯N, H⋯π and π⋯π interactions play a seminal role in generating fascinating supramolecular structures. Therefore, ON donor imines (*E*)-2-((4-phenoxyphenylimino)methyl)phenol (HL_1_), (*E*)-2-((4-(4-biphenyloxy)phenylimino)methyl)phenol (HL_2_), (*E*)-2-((4-(naphthalen-1-yloxy)phenylimino)methyl)phenol (HL_3_) and (*E*)-2-((4-(2-naphthoxy)phenylimino)methyl)phenol (HL_4_) (Scheme 4) have been prepared. The experimental results of brine shrimp cytotoxicity assays showed (lethal dose, 50%) LD_50_ values of <1 μg ml^−1^ exhibiting significant antitumor activity and with (half maximal inhibitory concentration) IC_50_ values of 14.20 and 4.54 μg ml^−1^, respectively. The biological findings showed significant activity in protecting DNA against hydroxyl free radicals in a concentration dependent manner. It is thought that these ligands at different doses could be used against different cancer-chemopreventive models and could seem to contrive safer medical treatments for future. In addition, the results indicated that the one-electron irreversible oxidation product is formed due to hydroxyl moiety and that the process is diffusion-controlled.^38^

**Scheme 5:**
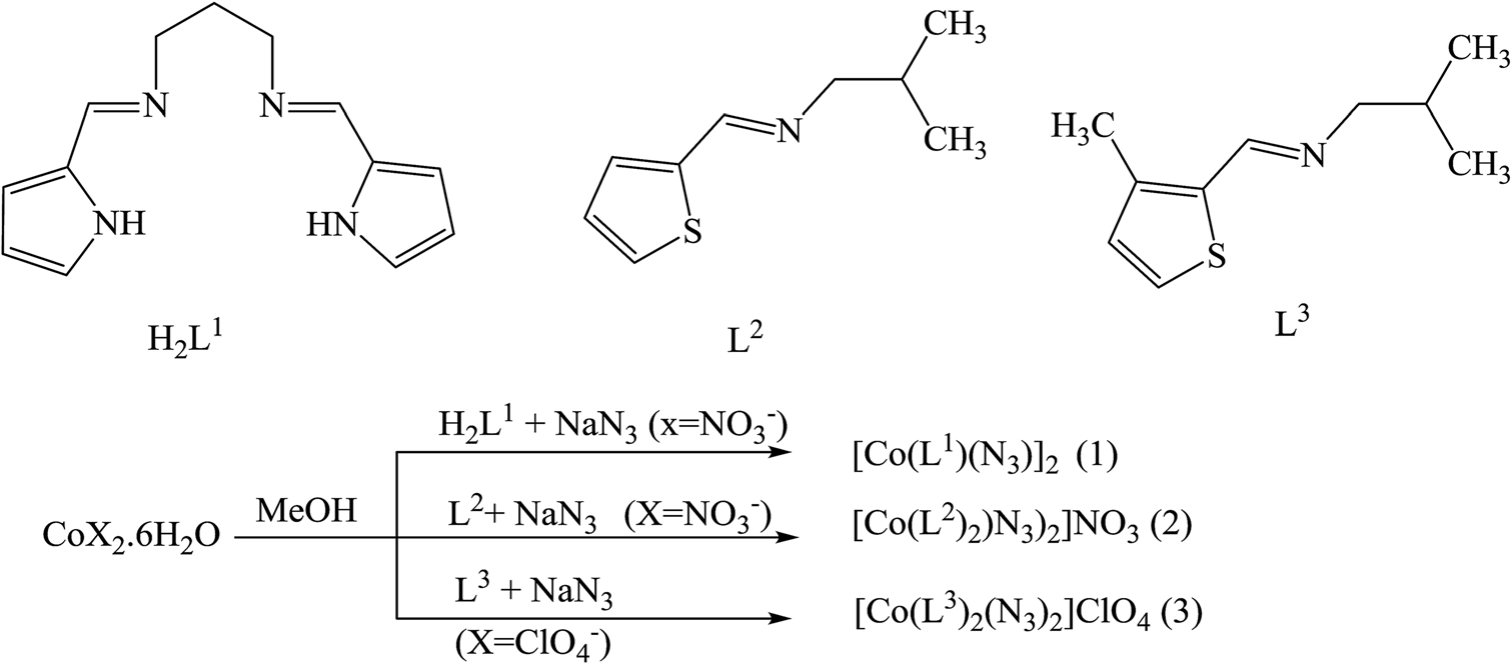
Structures of the ligands and the formation of complexes 1–3.

In addition, three SBs complexes of Co(iii), *viz.* [Co(L^1^)(N_3_)_2_] (1), [Co(L^2^)_2_(N_3_)_2_]NO_3_ (2), and [Co(L^3^)_2_(N_3_)_2_]ClO_4_ (3), where H_2_L^1^ = *N*,*N*′-propane-1,3-diylbis[1-(pyrrol-2-yl)methanimine], L^2^ = 1-[(thiophen-2-ylmethylidene)amino]propan-2-amine and L^3^ = 1-[(3-methylthiophen-2-ylmethylidene)amino]propan-2-amine (Scheme 5), have been prepared and characterized. The syntheses have been achieved by the reaction of Co(ii) ions with the tetradentate Schiff base (H_2_L^1^) or the bidentate ligands L^2^ and L^3^ in the presence of azide. Structural studies reveal that all of the complexes comprise a CoN_6_ chromophore in which the central Co(iii) ions adopt a distorted octahedral geometry. Whereas compound (1) is a binuclear end-on bis(μ-azido) complex, both compounds (2) and (3) are mononuclear with two terminal azide ions in *cis* positions. The antibacterial activity of all the complexes and their constituent Schiff base ligands has been tested against some Gram (+) and Gram (−) bacteria. From these results, it could be concluded that the previous compounds have mild to strong bactericidal properties which increase with amount to some extent. However, none of these compounds is as potent as commercial antibiotics like ciprofloxacin at similar concentrations. These results of the work certainly suggest that the antibacterial activity of the complexes has been shown by the nature of the constituent compounds.^39^

**Scheme 6:**
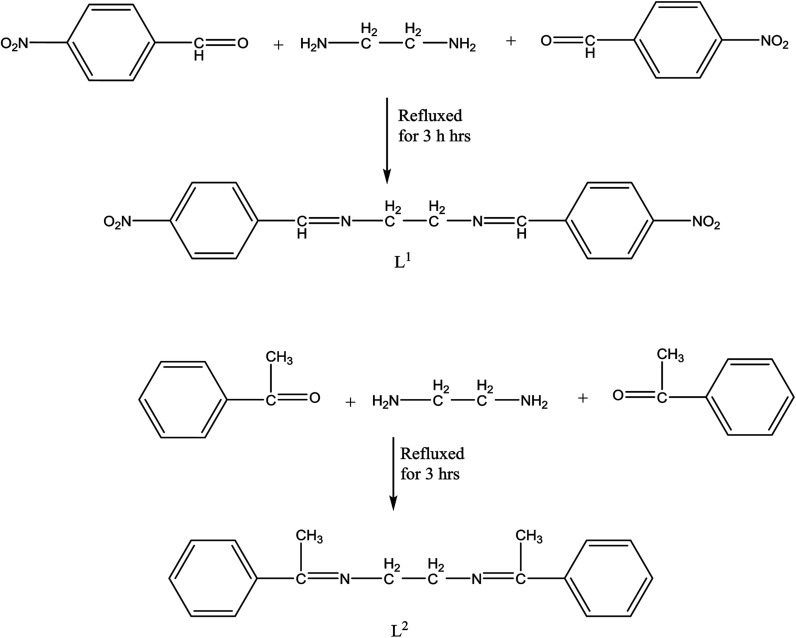
Structures of SBs (L^1^ and L^2^).

*N*,*N*-Bis(4-nitrobenzaldehyde)ethylenediamine (L^1^) and *N*,*N*-bis(acetophenone)ethylenediamine (L^2^) (Scheme 6) and its Zn(ii), Cd(ii) complexes have been prepared and studied in different physicochemical studies. The bioefficacy of SBs and their complexes have been evaluated against the growth of (*Staphylococcus aureus* and *Escherichia coli*) bacteria *in vitro* to assess their bioactivity potential. The improved activity of the metal complexes might be attributed to their increased lipophilic environment arising due to chelation. Besides, the increase in lipophilicity enhances the penetration of SBs and their metal complexes into the lipid membranes and thus restricts further growth of the organism. Also, the SBs and their metal complexes are more toxic to *S. aureus* than to *E. coli*, probably due to the sulfonic OH, OCH_3_, S and CH_3_CH_2_CH groups, which might interact with the double membrane. This activity is related to the nature and structure of the complexes.^40^

**Scheme 7:**
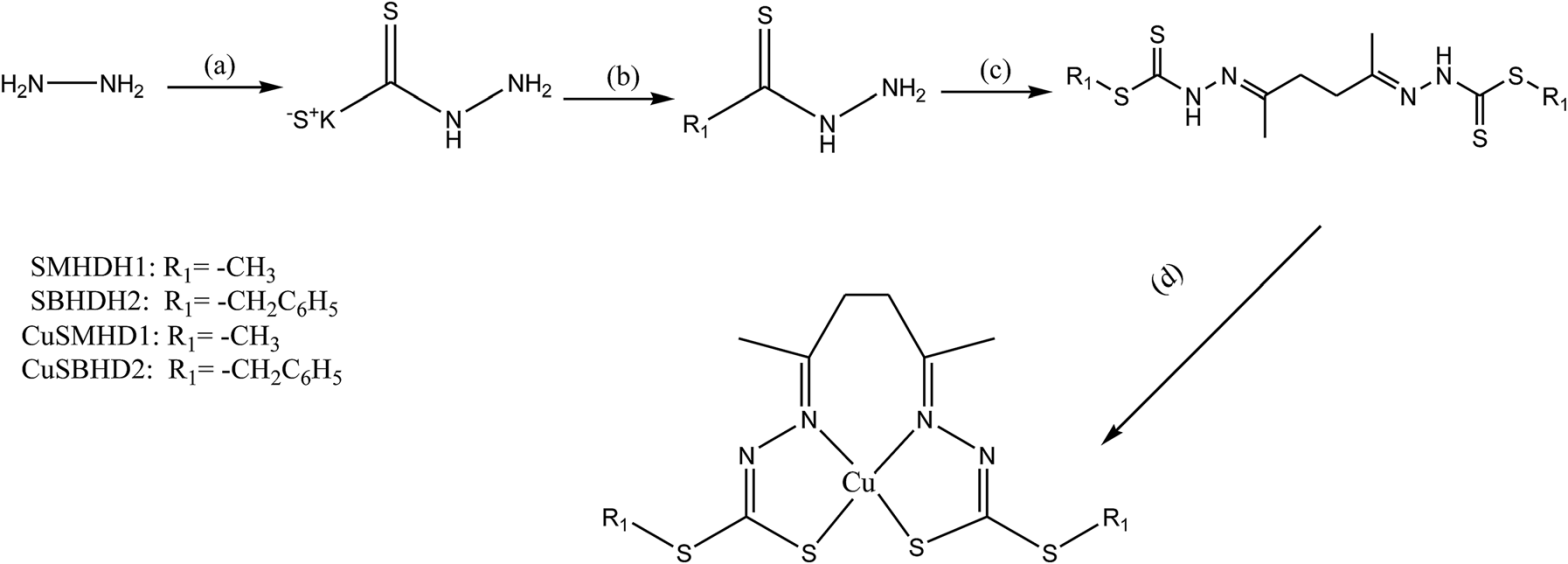
Synthesis of the copper complexes from bis(dithiocarbazate) ligands. *Reagents and conditions:* (a) CS_2_, KOH, EtOH, 0 °C, 1 h; (b) CH_3_I or PhCH_2_Cl, EtOH, 0 °C, 5 h; (c) for SMHDH_2_ (2,5-hexanedione, EtOH, 79 °C, 1 h), for SBHDH_2_ (2,5-hexaedione, EtOH, 79 °C, 5 min) and (d) for CuSMHD [Cu(OAC)_2_·H_2_O, MeOH, 65 °C, 1 h], for CuSBHD [Cu(OAc)_2_, acetonitrile, r.t, 1 h].

SB complexes containing copper ions reveal surprising molecular diversity, not only in coordination geometry but also in more subtle changes in the ligands. These ligands with copper ions represent a class of compounds with the most interesting properties, from the point of view of both chemical and biological behavior. In this regard, Cu(ii) complexes prepared from the condensation of *S*-methyl- and *S*-benzyl-dithiocarbazate with 2,5-hexanedione (SMHDH2 and SBHDH2 respectively) (Scheme 7) have been characterized using various physicochemical and spectroscopic procedures. SBs and their complexes were evaluated for their ability to hinder the growth of ten strains of Gram-negative and Gram-positive bacteria. Besides that, *antiproliferation* activity was found to be improved by complexation with Cu^2+^ ions. Moreover, initial screening showed copper complexes are powerfully active against human breast adenocarcinoma cancer cell lines MDA-MB-231 and MCF-7. In addition, SMHDH2 exhibited a broad range of moderate activity against various strains. It was most effective against *E. coli AcrAB*-, *Acinetobacter baumannii*, *Pseudomonas aeruginosa* and *S. aureus*, thus making it a potential antimicrobial agent in the presence of polymyxin B nonapeptide (PMBN). Moreover, the enhanced activity observed for SMHDH2 compared to its *S*-benzyl analog is consistent with the observation that the SB prepared from 2-benzoylpyridine with *S*-methyldithiocarbazate (SMDTC) was a highly effective inhibitor of *E. coli* and *S. aureus* whereas that prepared with the *S*-benzyldithiocarbazate (SBDTC) analog showed no activity.^41^

**Fig. 1:**
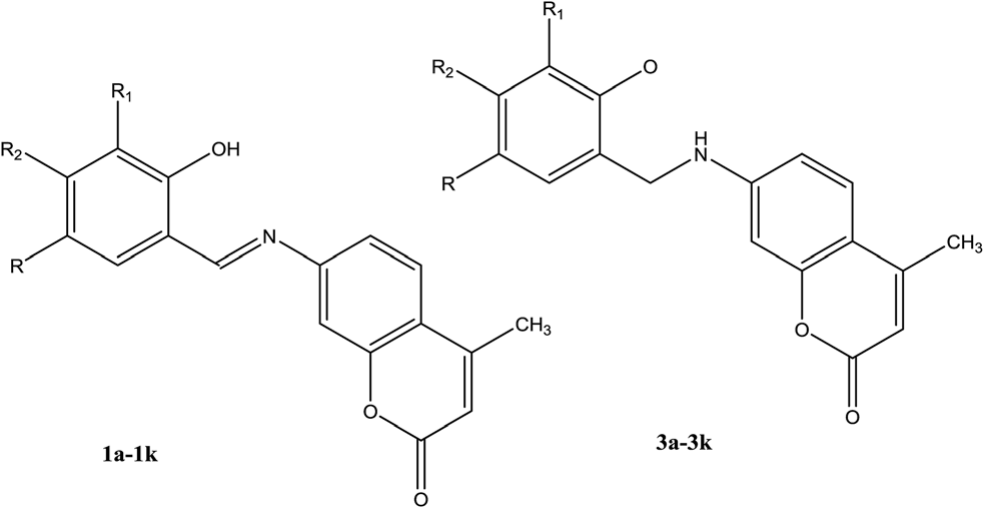
Structures of Schiff bases 1a–1k and their corresponding secondary amines 3a–3k showing the numbering system used in the assignment of ^1^H and ^13^C NMR spectra.

Creaven *et al.*^42^ have reported the preparation and screening of linezolid-like SBs as inhibitors of biofilm formation. These compounds in the presence the 2-chloroquinolinyl and 2-chloro-8-methylquinolinyl motif, respectively, displayed antibacterial activity superior to that of linezolid and were stronger when compared with ciprofloxacin. Cu(ii) complexes of Schiff base-derived coumarin ligands (Fig. 1) have shown good anti-*Candida* activity. Moreover, the more active complexes and their corresponding ligands were investigated in the presence of Cu(ii) in liquid and frozen solution by ESR spectroscopic methods. However, cytotoxicity investigations of the amines, together with the Cu(ii) complexes and their corresponding ligands, against human colon cancer and human breast cancer cells recognized the chemotherapeutic potential of SBs.^43,44^

**Scheme 8:**
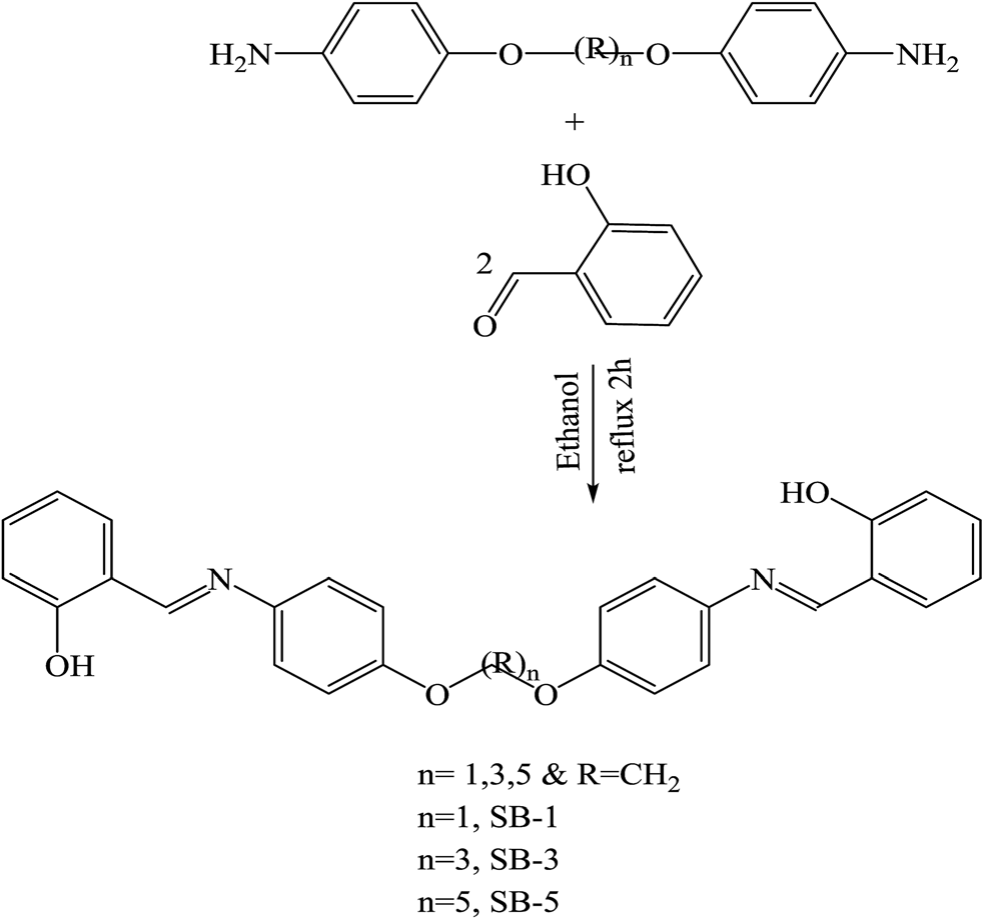
Synthesis of SB ligands.

On the other hand, new compounds from SBs were prepared by the reaction of salicylaldehyde with semiaromatic diamines which were synthesized by the reduction of the corresponding dinitro groups. For SB ligands (Scheme 8), their precursors and metal complexes were also examined for antibacterial, antitumor, brine shrimp lethality, antifungal, DPPH free-radical scavenging and DNA damage assays. The data of these analyses showed the essential potential of the prepared SBs, their precursors and copper(ii) complexes in the biological field as near future drugs.^45^

#### 2.1.2 Heterocyclic Schiff bases

**Scheme 9:**
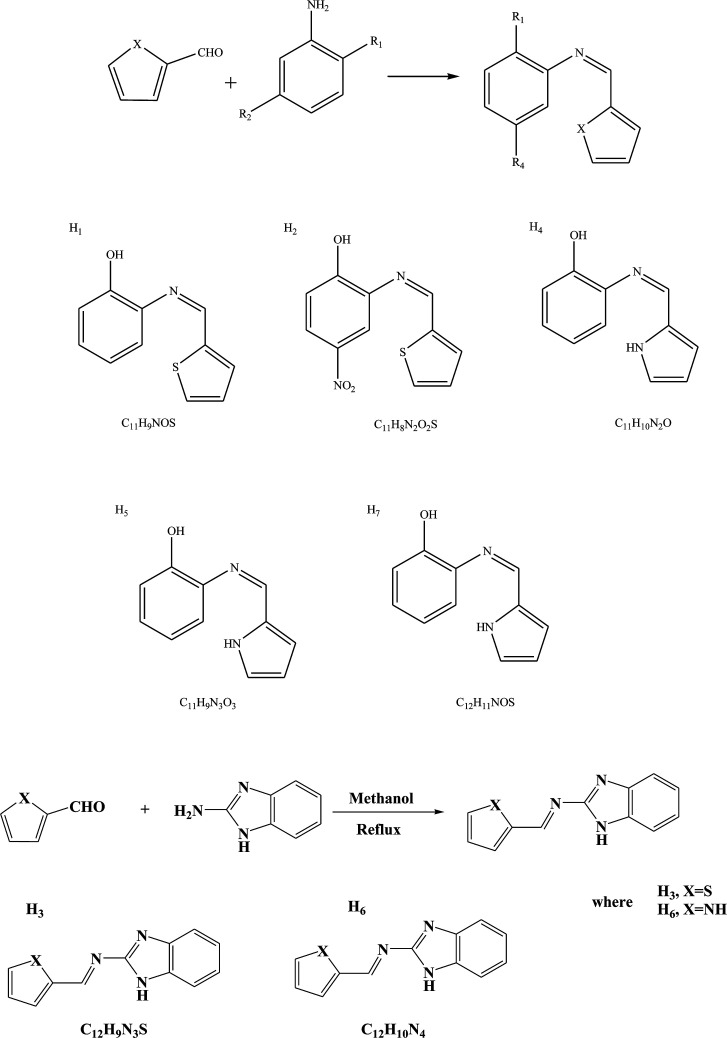
Preparation of Schiff bases (SBs).

SBs containing heterocyclic moieties deserve special attention because of their significant role in biological processes as a very good chemotherapeutic agent and also an important antioxidant chemical.^45,46^ Their role in the removal of toxic chemicals from biological systems is excellent. The current literature search has highlighted antibacterial resistance in community pathogens as a major problem, and to overcome this a new generation of antibiotics should be revived.^47,48^ For instance, Shanty *et al.* have prepared Schiff bases (H_1_–H_7_) (Scheme 9). These compounds were screened for enzyme inhibition, antibacterial, cytotoxic and *in vivo* antidiabetic activities and compounds were found to be active against one or other activity. The findings indicated that Zn(ii) complexes are a good inhibitor of alkaline phosphatase enzyme and possess the highest potential against diabetes, blood cholesterol levels and cancer cells. In other words, the difference in the effectiveness of SBs against different organisms depends either on the differences in the ribosomes of microbial cells or the impermeability of the cells of the microbes. Besides that, the inhibition of microbial growth was not observed with the *E. coli*, possibly due to the presence of an outer protective layer called lipopolysaccharide.^49^ On the other hand, some SBs have been prepared by the condensation of 2-aminophenol, 2-amino-4-nitrophenol, 2-amino-4-methylphenol, and 2-aminobenzimidazole with thiophene-2-carboxaldehyde and pyrrole-2-carboxaldehyde. SBs individually exhibited varying degrees of inhibitory effects on the growth of the tested bacterial species. Form these works, compounds containing electron-donating groups (OH) show moderate activity against the bacteria *Salmonella* Typhi, *Bacillus coagulans*, *Bacillus pumills*, *Clostridium* and good activity against *Bacillus circulans*. Moreover, SBs containing electron-acceptor groups (NO_2_) did not exhibit any significant effect against the same bacterial strains.^50^

**Scheme 10:**
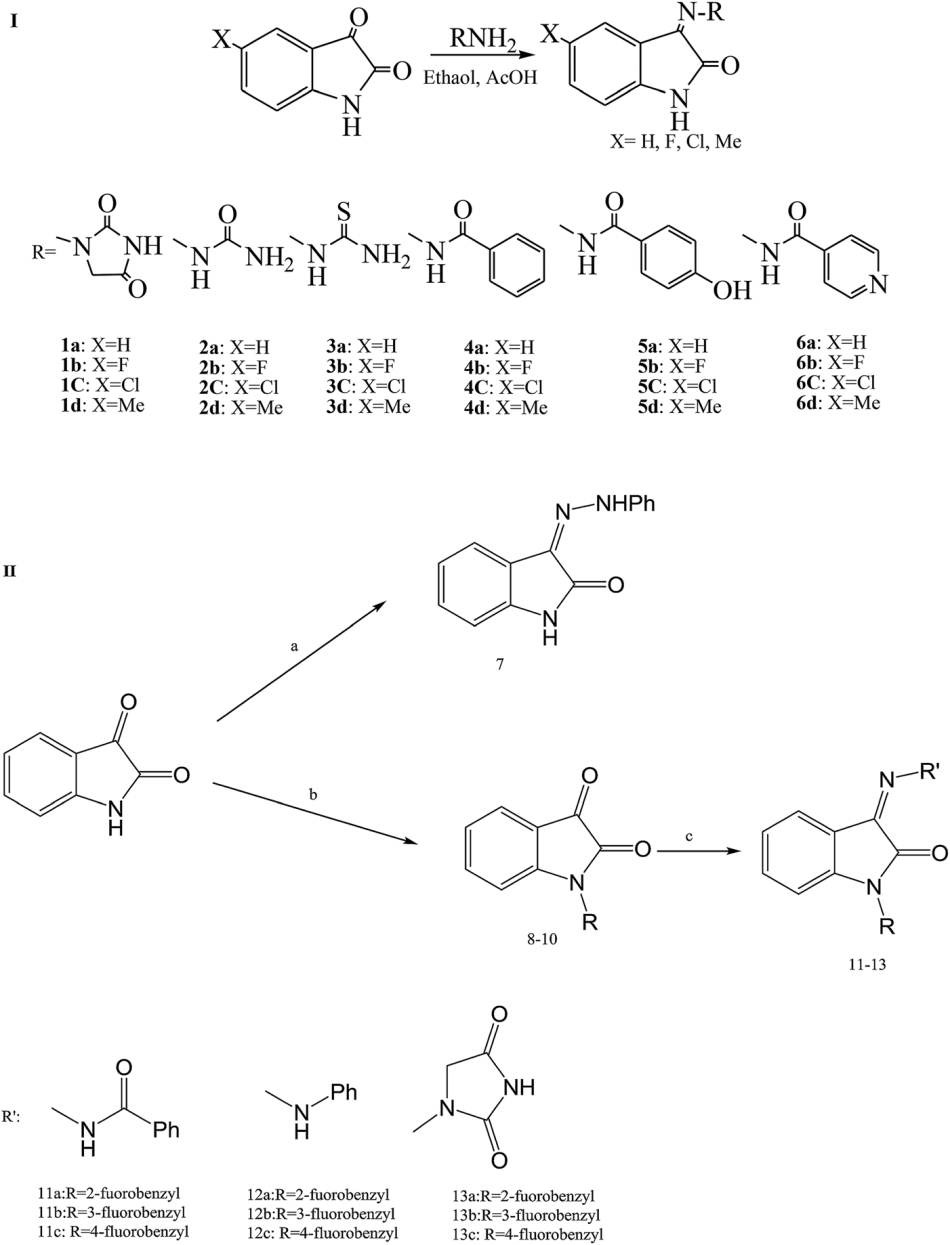
(I) Synthesis route to Schiff bases of 5-substituted isatins. (II) Synthetic routes to compound 7 and Schiff bases of *N*-arylmethylisatin 11a–13c. *Reagents and conditions:* (a) phenylhydrazine, ethanol 96% (w/w), acetic acid; (b) fluorinated benzyl chlorides, K_2_CO_3_, KI, acetonitrile; (c) compounds 8–10, ethanol 96% (w/w), acetic acid.

As in other modified isatin compounds, SBs have been synthesized by reacting 5-substituted isatins with bioactive compounds from amines or hydrazides. This work examined the effect of isatin *N*-substitution on the bioactivity of the resulting SBs (Scheme 10). Compounds containing functional groups CH_3_ (2d), F (3b), Cl (5c) and CH_3_ (6d) were among the most potent derivatives against *P. aeruginosa* (MIC (minimum inhibitory concentration) = 6.25 mg mL^−1^). This is an important result since *P. aeruginosa* is a highly opportunistic pathogen and SBs with activity against this *pathogenic bacterium* are of singular assessment. Analysis of the structure–activity relationship showed that the incorporation of (SH) urea-based Schiff bases leads to stronger derivatives with a broader spectrum of antibacterial activity. In addition, highly lipophilic compounds such as 11a–12c did not show any measurable antibacterial activity, which suggests that an optimal lipophilicity might be an important requirement for the antibacterial activity of the studied isatins.^51^

**Scheme 11:**
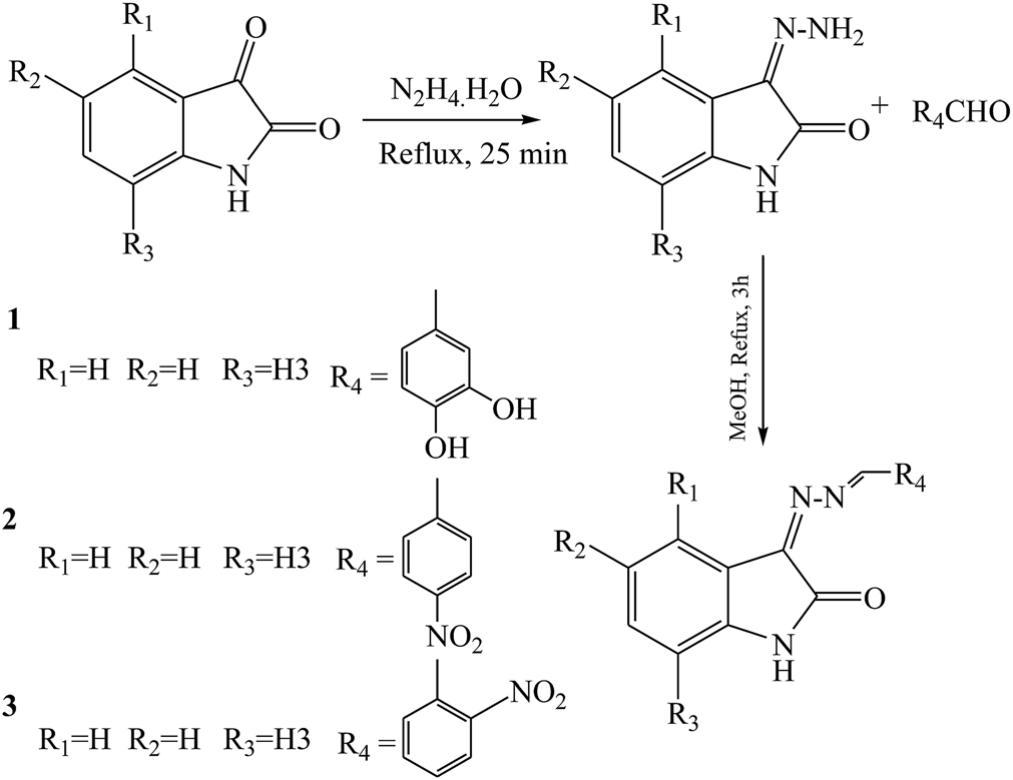
Synthesis of bis-Schiff from isatins.

Khan *et al.*^52^ have reported the synthesis and evaluation for *in vitro* antiglycation of bis-Schiff bases (Scheme 11). Some of these bis-Schiff bases such as compounds 1 (IC_50_ (degree of antiglycation activity) = 291.14 ± 2.53 IM), 2 (IC_50_ = 257.61 ± 5.63 IM), and 3 (IC_50_ = 243.95 ± 4.59 IM) showed an admirable antiglycation activity, better than the normal (rutin, IC_50_ = 294.46 ± 1.50 IM). A remarkable effect on the antiglycation activity was exhibited owing to the presence of electron-withdrawing groups at isatin. Therefore, this difference in activity might be due to the presence of the Cl group.

**Scheme 12:**
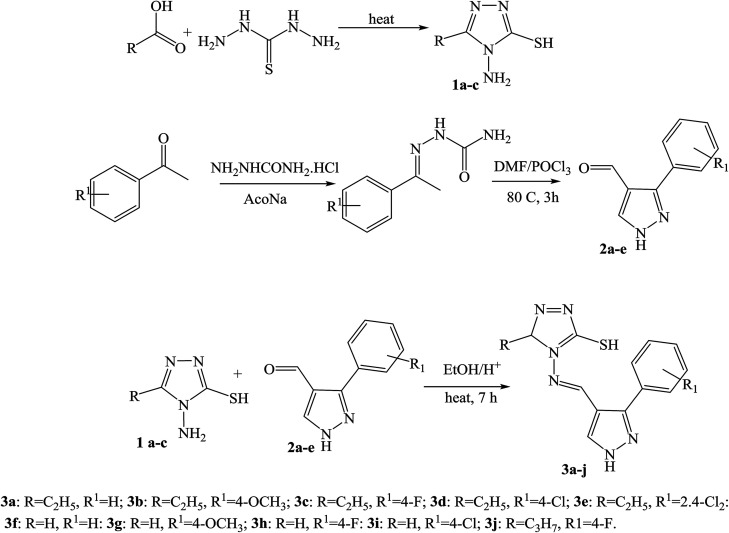
Synthetic route for SBs.

Moreover, a series of new pyrazole-based SBs (Scheme 12) were synthesized and characterized by spectral analyses. Prepared compounds were evaluated for their antibacterial activity against *Bacillus subtilis*, *Staphylococcus aureus*, *Pseudomonas aeruginosa*, and *Escherichia coli*. As a result, compound 3f was shown to have admirable activity against *S. aureus* compared with a standard against the remaining three microorganisms.^53^

**Scheme 13:**
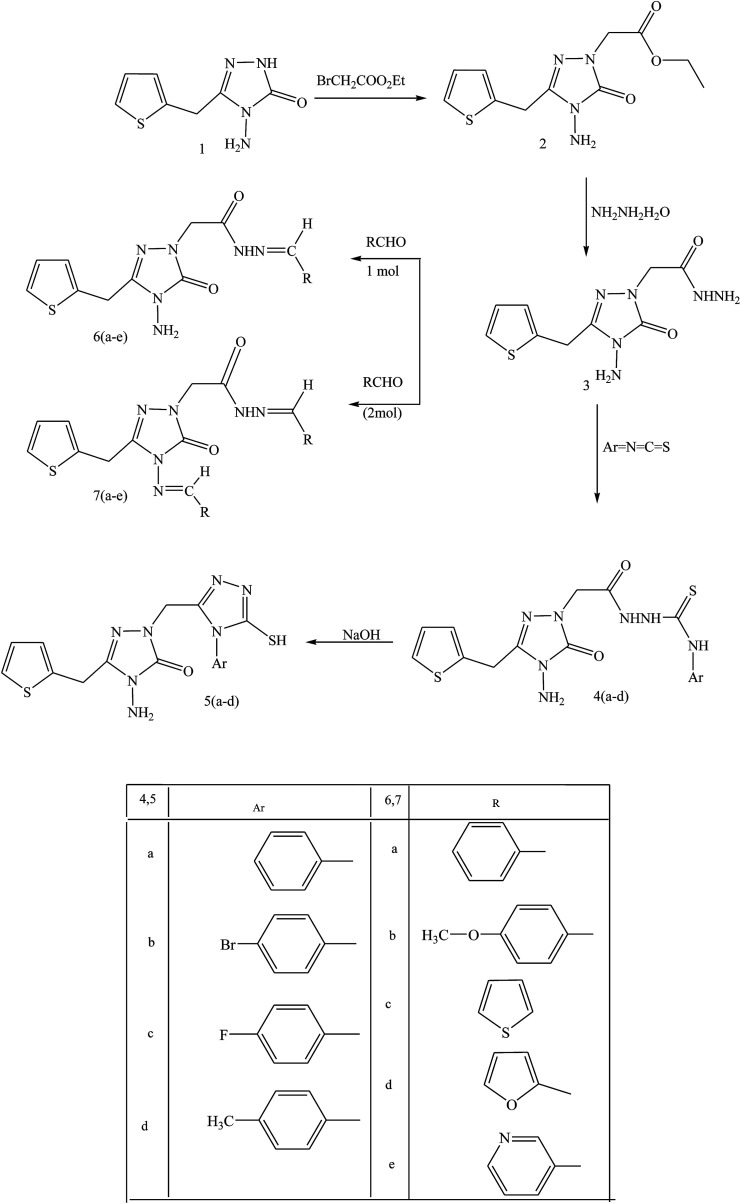
Synthetic pathway for the protection of target compounds 2, 3, 4, 5, 6 and 7.

In this same review, essential compound 2-(4-amino-5-oxo-3-(thiophene-2-ylmethyl)-4,5-dihydro-1,2,4-tiazole-1-yl) acetohydrazide (3) was prepared by reacting hydrazine hydrate with ethyl-2-(4-amino-5-oxo-3-(thiophene-2-ylmethyl)-4,5-dihydro-1,2,4-triazole-1-yl) acetate (2), obtained in basic medium from 4-amino-5-(thiophene-2-ylmethyl)-2*H*-1,2,4-triazole-3(4*H*)-one (1). Compound 3 was converted to the thiosemicarbazide and SB derivatives (Scheme 13). As with results for bioactivity, the thiosemicarbazide derivatives also exhibited antibacterial activity against *Bacillus cereus*, *Staphylococcus aureus*, and *Mycobacterium smegmatis*. Moreover, the thiosemicarbazide group deserves deliberation in the synthesis of bioactive Schiff bases. The reason for Gram (−) bacteria [*Escherichia coli*, *Yersinia pseudotuberculosis*, and *Pseudomonas auroginosa*] being resistant to the compounds tested may be due to the relatively more important structure of the cell wall including the more closely spaced peptidoglycan and extra lipopolysaccharide layers.^54^

In continuation of these fields on Schiff bases and their complexes, several works have been reported on the synthesis and characterization of Schiff base ligands and their complexes: 3-amino-4-{1,5-dimethyl-3-[2-(5-methyl-1*H*-indol-3-yl)-ethylimino]-2-phenyl-2,3-dihydro-1*H*-pyrazol-4-ylazo}phenol^54^ and 3-[2-(1*H*-indol-3-yl)-ethylimino]-1,5-dimethyl-2-phenyl-2,3-dihydro-1*H*-pyrazol-4-ylamine, 1-{[2-(1*H*-indol-3-yl)-ethylimino] methyl}naphthalene-2-ol and dicyclohexyl amine and 4-((2-hydroxy-1-naphthyl)methylene amino)-1,5-dimethyl-2-phenyl-1*H*-pyrazol-3(2*H*)-one (HL).^55–57^ The previous ligands and their metal complexes were screened for their biological activity against bacterial species, two Gram-positive bacteria (*B. subtilis* and *S. aureus*) and two Gram-negative bacteria (*E. coli* and *P. aeruginosa*). In addition, the influence of the metal ion of the complexes upon the antibacterial activity against the tested Gram-positive and -negative organisms shows that the complexes have an enhanced activity compared to the ligand itself. Such increased activity of the complexes can be explained on the basis of Tweedy’s chelation theory.^58^ Chelation reduces the polarity of the metal ion considerably because of the partial sharing of its positive charge with the donor group and also due to p-electron delocalization on the whole chelate ring.

#### 2.1.3 Ferrocene-based Schiff bases

**Scheme 14:**
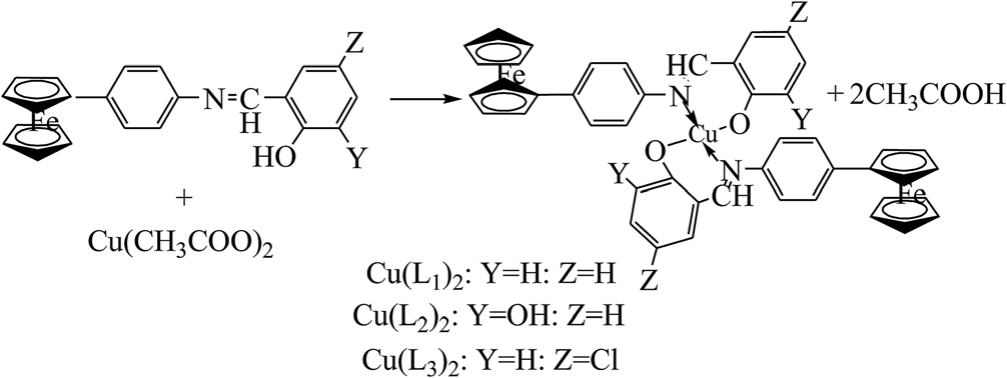
Synthesis of copper complexes [Cu(L_1_)_2_–Cu(L_3_)_2_].

Ferrocene-based organometallics and metal complexes possess unique properties like stability, aromaticity, redox activity, lipophilicity and different membrane permeation. Ferrocene containing metal–ligand complexes can be regarded as multinuclear molecules possessing the features of both coordination chemistry and of organometallics.^59^ Moreover, ferrocenyl Schiff bases derived from aryl amines and their complexes show potent antitumor and DNA-protecting activity due to their interesting electrochemical properties, thus making them an attractive pharmacophore for drug design.^60–63^ For instance, three ferrocene-based SB Cu(ii) complexes, *i.e.* bis(*N*-(2-hydroxybenzylidene)-4-ferrocenylaniline) copper(ii) (Cu(L_1_)_2_), bis(*N*-(2,3-dihydroxybenzylidene)-4-ferrocenylaniline) copper(ii) (Cu(L_2_)_2_), and bis(*N*-(5-chloro-2-hydroxybenzylidene)-4-ferrocenylaniline) copper(ii) (Cu(L_3_)_2_) (Scheme 14), were prepared from ferrocenyl SBs. The synthesized SBs and their Cu(ii) complexes were characterized by various spectroscopic and electroanalytical methods. Moreover, the SBs exhibited significant activity in brine shrimp cytotoxicity and antitumor assays with IC_50_ values ranging from 2.32 to 69.61 mg ml^−1^. Also, the SB complexes were found to be more active than their ligands with lower IC_50_ values. A DNA–drug interaction study through voltammetry revealed their binding nature which complemented the antitumor behaviour evaluated from biological studies.^64^ This interaction is logically acceptable due to the presence of Fe^2+^ in the form of the ferrocene moiety in the solution of compound which can interact with the negatively charged phosphate groups of DNA.

**Scheme 15:**
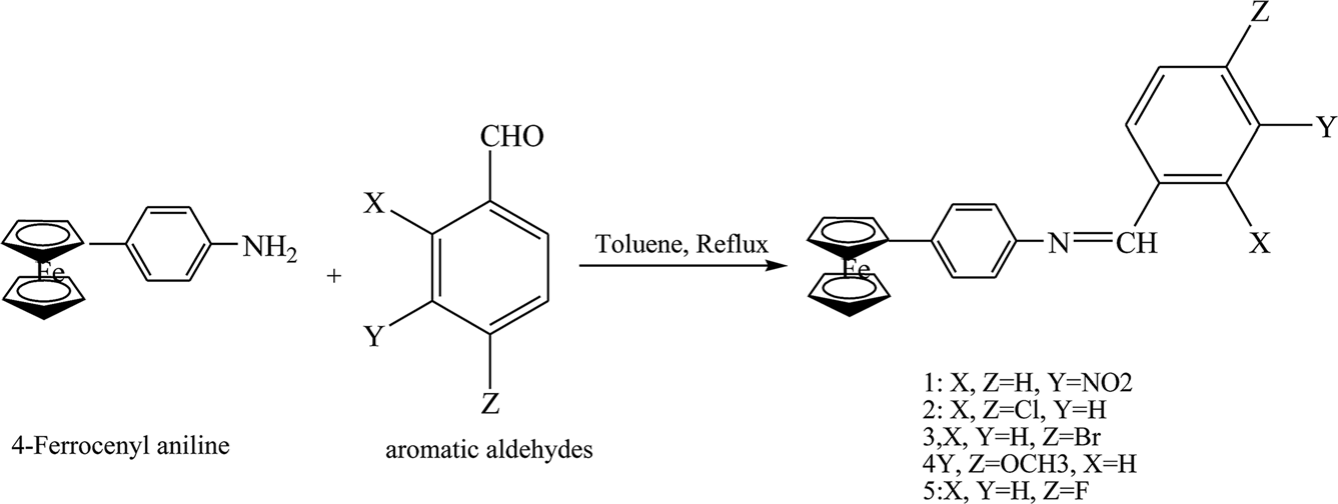
Synthesis of Schiff bases 1–5.

Zaheer *et al.*^65^ have reported the synthesis of ferrocene SBs (1–5) by the condensation of 4-ferrocenyl aniline with different substituted aromatic aldehydes and acetyl acetone (Scheme 15). These compounds showed low cytoxicity and appreciable antifungal, antioxidant and DNA-protection activities due to the low solubility of Schiff bases in DMSO.

#### 2.1.4 C6-Schiff bases

**Scheme 16:**
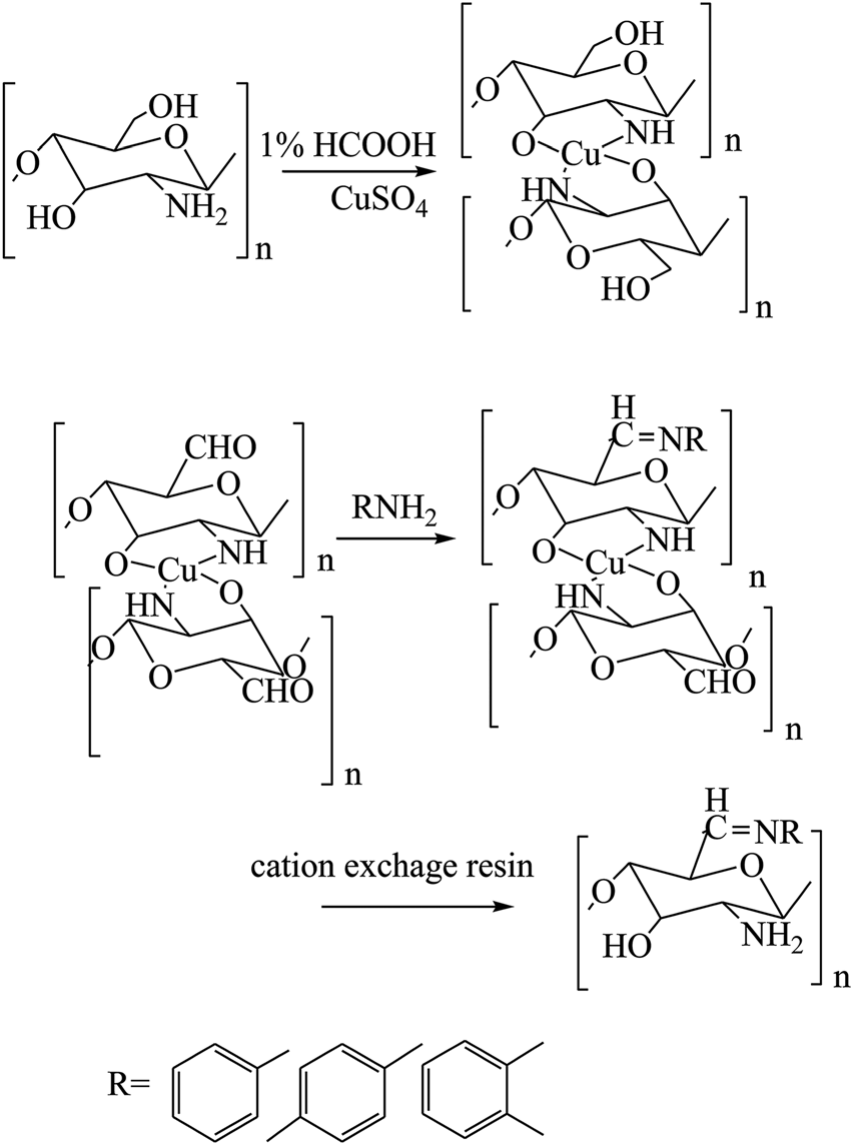
Synthesis of C6-Schiff bases of chitosan derivatives.

**Scheme 17:**
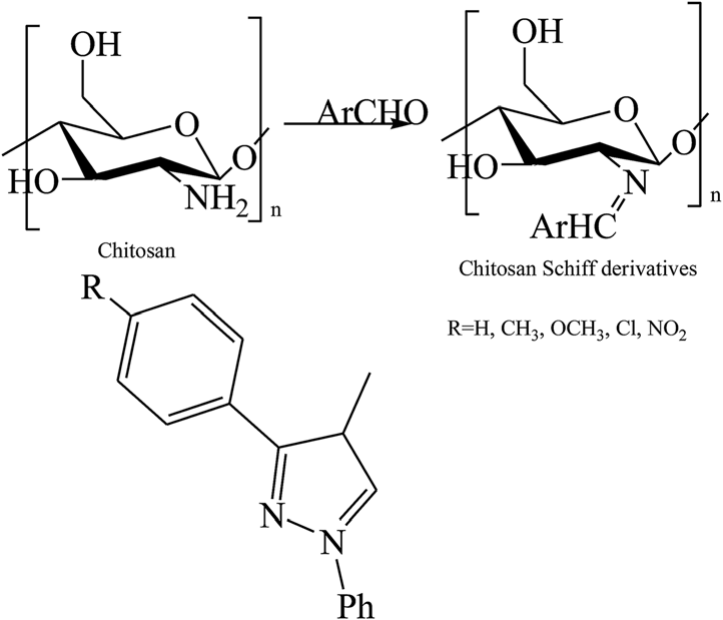
Synthesis of chitosan Schiff bases (ChBs).

**Scheme 18:**
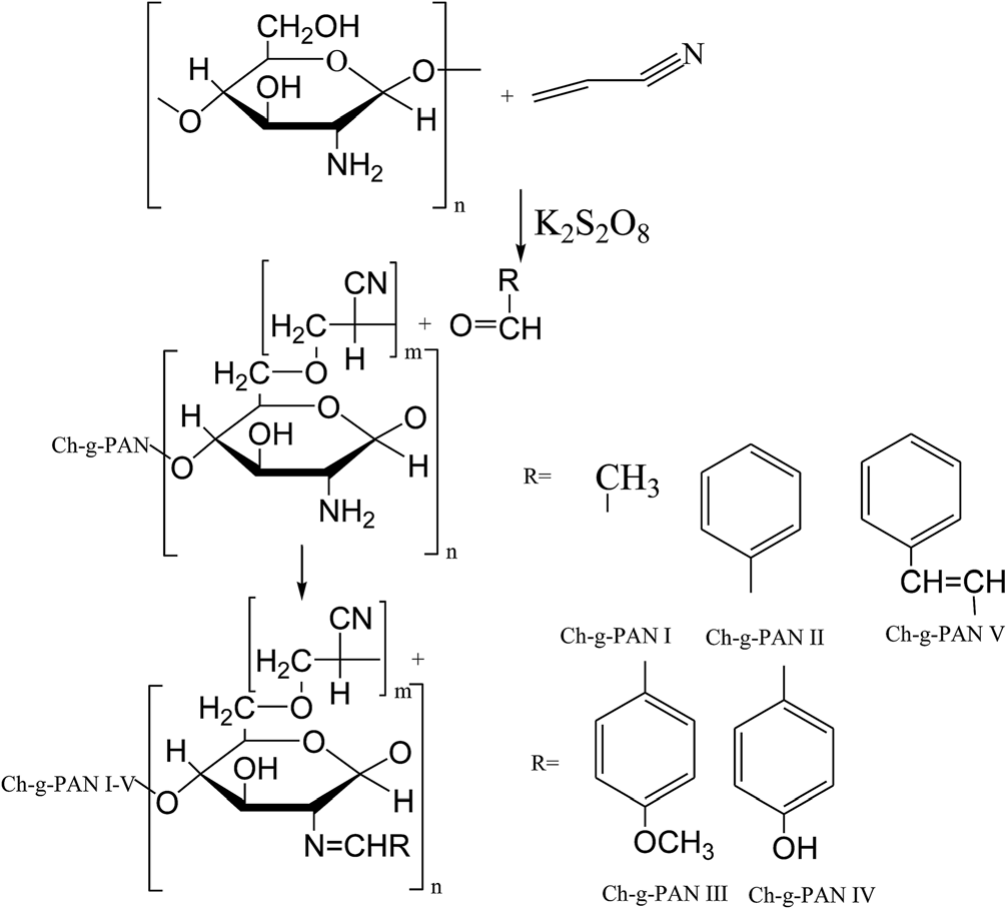
Modification of chitosan-graft-polyacrylonitrile.

To further enhance the antimicrobial activities of chitosan, three types of different C6-Schiff base derivatives (Scheme 16) of chitosan have been synthesized in the deprotection of C_2_–NH_2_ with cation exchange resin. Therefore, the antibacterial activities of the compounds were tested in the research, and experimental data exhibited that the chitosan derivatives had considerably improved antibacterial activity; therefore, the results indicated that the antimicrobial activity of the SBs was stronger than that of chitosan and was dependent on the substituent groups (Scheme 17). On the other hand, another work has been published on the graft copolymerization of chitosan with acrylonitrile prepared by free radical polymerization using potassium persulfate (K_2_S_2_O_8_) as the initiator (Scheme 18). The results of antibacterial activity of the original chitosan exhibited that the antimicrobial activity increased with the increase in percentage grafting, so the modified SB was carried out on the highly grafted Ch-g-PAN at 155%. This was due to the interaction between the positively charged chitosan molecules and the negatively charged microbial cell membrane. The interaction was mediated by electrostatic forces between the protonated –NH^+^ groups of chitosan and the electronegative charge on the microbial cell surface.^66–68^

**Scheme 19:**
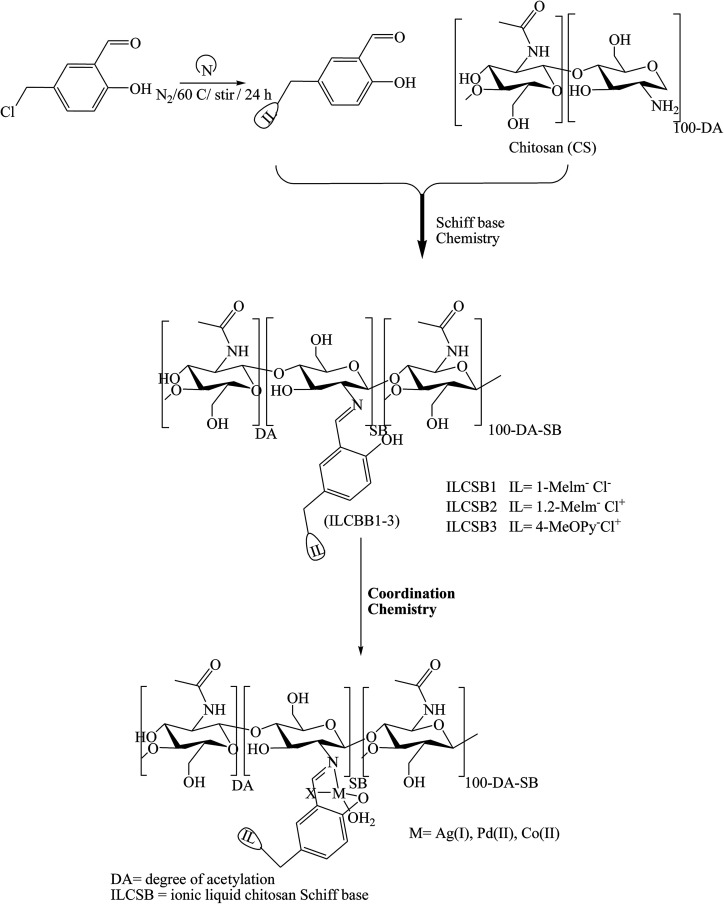
Schematic representation of ionic liquid-anchored chitosan Schiff bases and their metal complexes.

Some attempts to develop a new category of medical candidates with antimicrobial and anticancer efficacy in a series of SBs bearing salicylidene ionic liquid (IL-Sal) brushes (ILCSB1-3, poly-(GlcNHAc-GlcNH_2_-(GlcN-Sal-IL))) (Scheme 19) was effectively prepared by adopting efficient synthetic paths. In conclusion, the functionalization of chitosan with IL-Sal brushes together with metalation of the formed ILCSBs synergistically improved its antimicrobial and antitumor features to a great extent.^69^

### 2.2. Schiff bases as corrosion inhibitors

Schiff bases used as inhibitors may block anodic, cathodic or both sites, thus preventing the metallic substrate from undergoing hydrogen evolution reactions or metal dissolution with a predominance of film-developing surface adsorption. The corrosion inhibition efficiency of organic inhibitors has been reported to be shown by a strong affinity for inorganic surfaces, with low influences on the atmosphere.^70^ They build up protective hydrophobic film molecules adsorbed on the metallic surface, thus providing a barrier to the dissolution of the substrate in the electrolyte. The inhibitory action of SBs has been described by a well-recognized model,^71–73^ which involves the adsorption of SBs on the corroding metal surface with displacement of adsorbed water molecules from the metal surface as represented by the following eqn (1):

1 SB_(sol)_ + *a*H_2_O_(ads)_ ↔ Org_(ads)_ + *a*H_2_O_(sol)_

The four main modes of adsorption associated with inhibitor organic molecules at metal surfaces include (I) electrostatic interaction of the corrosion inhibitor (CI) with the metal surface, (II) charge sharing between the metal and the CI, π-back bonding, and organometallic complex formation.^74^ Furthermore, (III) the stability of the adsorbed organic molecule films/layer on the substrate surface depends on the nature of the functional groups, possible steric factors, aromaticity, electron density of the donor atoms, the type of corrosive solution and nature of the interaction between its (IV) p-orbital with the d-orbital of the metal. The inhibitory efficiency of these SBs is related to the structural nature of the SBs with N, S, O and P heteroatoms in the molecules, which serve as reaction cores for physical adsorption on the substrate surface.^75^ The inhibition efficiency of the heteroatoms in SBs follows the sequence O < N < S < P.^76,77^ The transfer of electrons from these compounds to the substrate surface is facilitated by the availability of lone pairs and π-electrons in the organic molecules, leading to formation of coordinate covalent or non-covalent bonds with the metals. The strength of the physical bond, and thus the performance of the SBs depend on the electric charge density on the donor atom of the functional group, the polarizability of the group and the electronic and stereo structure of the organic molecules. The inhibition action could be due to the adsorption of the organic molecules or its ions on anodic and/or cathodic sites, an increase in the cathodic and/or anodic overpotential, and the ability to form a protective barrier film. Some of the issues that could influence inhibition action include the chain molecular/length size, bond strength, conjugated/aromaticity bonding, number and type of bonding atoms in the molecule, cross-linking ability and solubility in the environment. However, with increasing chain length of the hydrocarbon, there is a tendency to observe a diminishing in the corrosion inhibition performance due to the decreasing solubility in aqueous solution. The inhibition performance could be improved if the hydrogen atom attached to the carbon in the ring is replaced by electron-donating substituents such as –CHO, –NH_2_, or –CO_2_H.^78^ This will lead to changes in electron density in the metal at the point of attachment, resulting in retardation of the cathodic or anodic reaction, thereby minimizing corrosion. Here, we focused on anti-corrosion of some metals such as mild steel and aluminium using Schiff bases. Details on these metals are given below.

#### 2.2.1 Mild steel

**Scheme 20:**
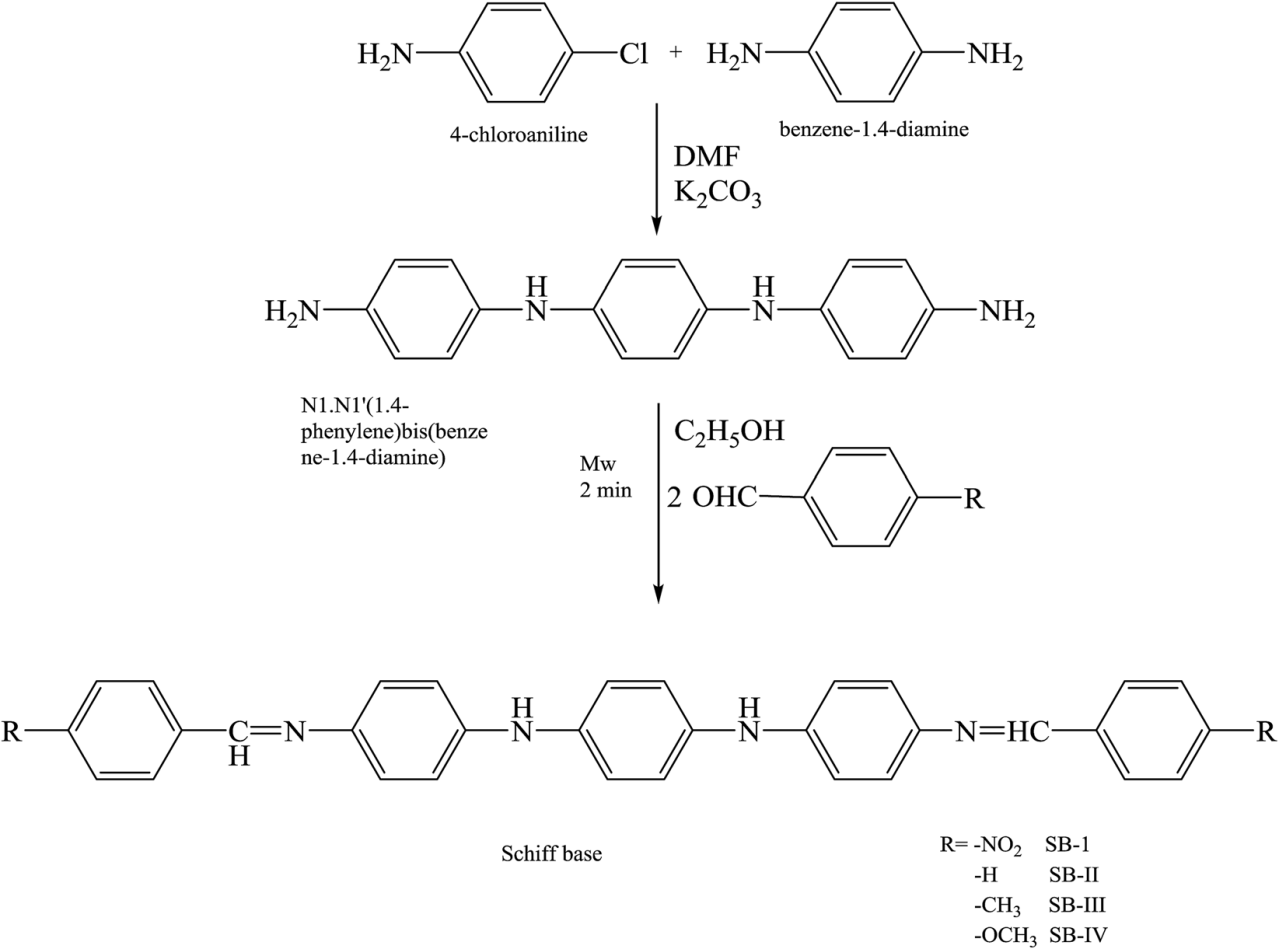
Synthetic route for the preparation of SBs.

Some works have reported the protective evaluation of four Schiff bases, namely *N*1,*N*1′-(1,4-phenylene)bis(*N*4-(4-nitrobenzylidene)benzene-1,4-diamine) SB-I, *N*,*N′*-(1,4-phenylene)bis(*N*-benzylidenebenzene-1,4-diamine) SB-II, *N*,*N*′-(1,4-phenylene)bis(*N*4-(4-methylbenzylidene)benzene-1,4-diamine) SB-III, and *N*1,*N*1′-(1,4-phenylene)bis(*N*4-(4-methoxybenzylidene)benzene-1,4-diamine) SB-IV (Scheme 20) for mild steel in 1 M HCl. The corrosion efficiency of SBs for mild steel is 71.42%, 89.52%, 92.85% and 96.19% for SB-IV, SB-I, SB-II and SB-III, respectively. Electrochemical analysis revealed that all of the SBs behaved as mixed-type inhibitors but predominantly of a cathodic type.^79^ The presence of the inhibitor causes a decrease in the corrosion current (*i*_corr_). The highest reduction in *i*_corr_ was obtained for SB-IV. This decrease in corrosion current is due to the increase in blocked fraction of a metal surface by physical adsorption and the order of inhibition efficiency is a follows: SB-IV > SB-III > SB-II > SB-I.

**Fig. 2:**
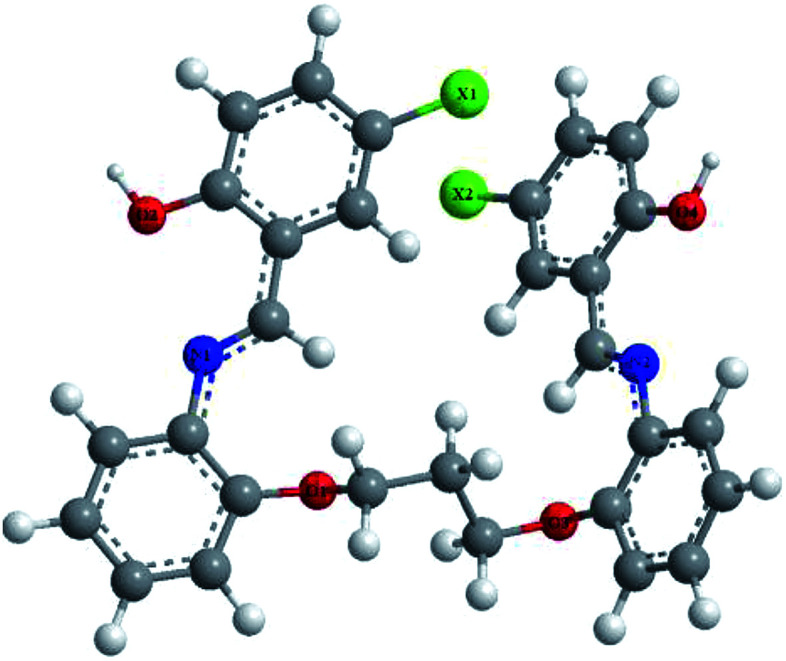
Molecular structure of investigated SBs (P1: X = H; P2: X = Cl; P3: X = Br).

**Fig. 3:**
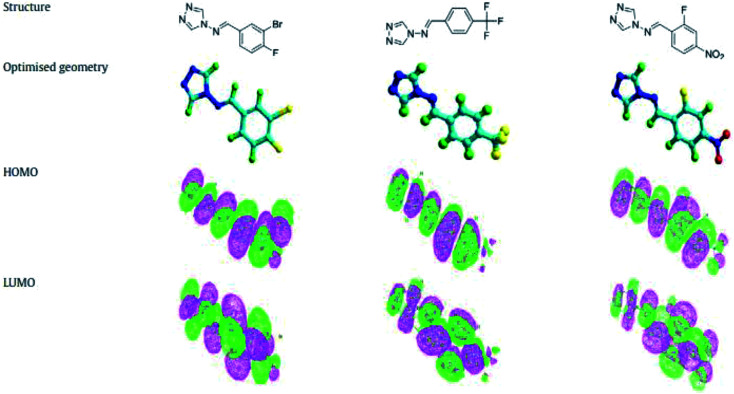
Optimized geometrical configurations of BFBT, TMBT, and FNBT.

Another study investigated the effect of three SBs, namely, 1,3-bis[2-(2-hydroxybenzylidenamino)phenoxy]propane (P1), 1,3-bis[2-(5-chloro-2-hydroxybenzylidenamino)phenoxy]propane (P2), and 1,3-bis[2-(5-bromo-2-hydroxybenzylidenamino)phenoxy]propane (P3) (Fig. 2), on the anti-corrosion of mild steel using electrochemical tests. Polarization measurements suggest that P1 acts as mixed-type inhibitor (synergistic effect) whereas P2 and P3 behave as mainly cathodic inhibitors for the acidic corrosion of steel. Electrochemical measurements show that inhibition efficiencies increase with an increase in the concentration of the organic compound. This reveals that the inhibitive actions of SBs were mainly due to adsorption on the metal surface. Adsorption of these organic compounds follows the Temkin adsorption isotherm. On the other hand, the results indicate that P2 and P3 are shifted to a negative direction; this effect is more evident at higher concentrations.^80^ The difference in protection activities of the reported inhibitors could be assigned to the presence of the electron-donating groups such as Br or Cl. These data show that the presence of Cl and Br atoms on the aromatic ring in P2 and P3 decreases the electronic density on the imine (C=N) group, oxygen and also the aromatic ring, which are responsible for adsorption on the metal surface. On the other hand, some authors have reported the investigation of triazole SBs (3-bromo-4-fluoro-benzylidene)-[1,2,4]triazol-4-yl-amine (BFBT), (4-trifluoromethyl-benzylidene)-[1,2,4]triazol-4-yl-amine (TMBT) and (2-fluoro-4-nitro-benzylidene)-[1,2,4]triazol-4-yl-amine (FNBT) (Fig. 3) as corrosion inhibitors on mild steel in 0.5 M HCl by chemical and electrochemical techniques. They found that the change in *E*_corr_ is less than ±85 mV, so the studied inhibitors are neither anodic nor cathodic but of the mixed type. This means that even though the kinetics of both metal dissolution and hydrogen evolution are altered by the addition of SBs, the one which is going to be more affected is the reduction of iron.^81^

**Fig. 4:**
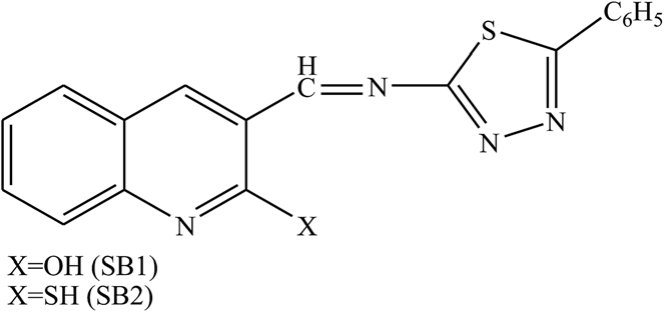
(((5-Phenyl-1,3,4-thiadiazol-2-yl)imino)quinolone-2-ol/thiol).

Schiff bases (Fig. 4) derived from heterocyclic amines and aldehydes have been prepared and evaluated as organic inhibitors for metal in 1 M H_2_SO_4_ by electrochemical measurements. In addition, the corrosion current density (*i*_corr_) values decrease with an increase in SB concentration suggesting the effectiveness of SBs as organic inhibitors, therefore, *E*_corr_ values are slightly shifted towards the negative direction and the Tafel constants *b*_a_ and *b*_c_ are changed with an increase in concentration of the inhibitor, suggesting that both the anodic dissolution and cathodic hydrogen evolution mechanism are affected in the presence of the inhibitor.^82^

**Fig. 5:**
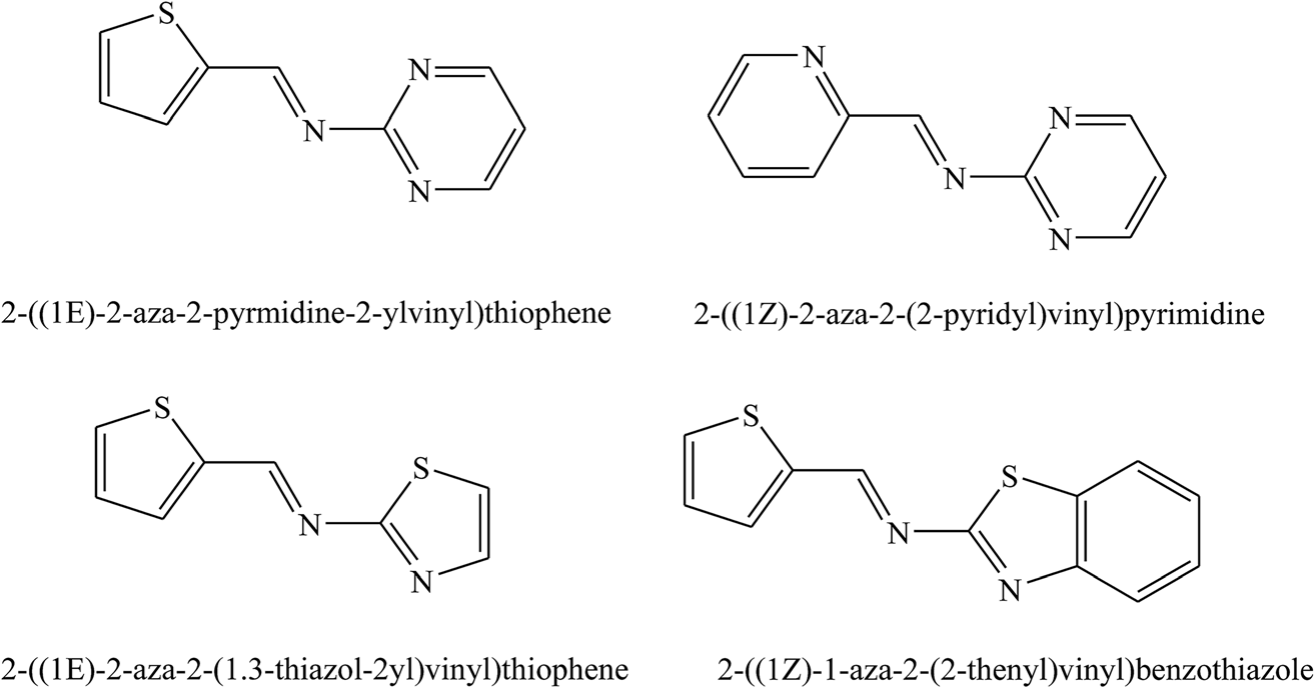
Structures of studied Schiff bases.

**Fig. 6:**
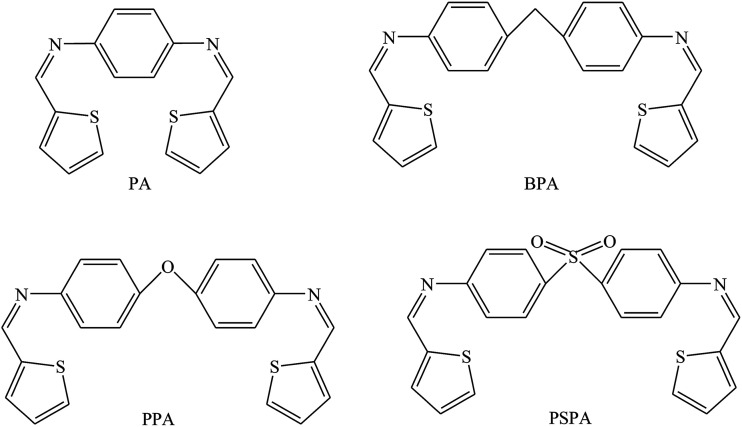
Chemical structure of the investigated compounds.

**Fig. 7:**
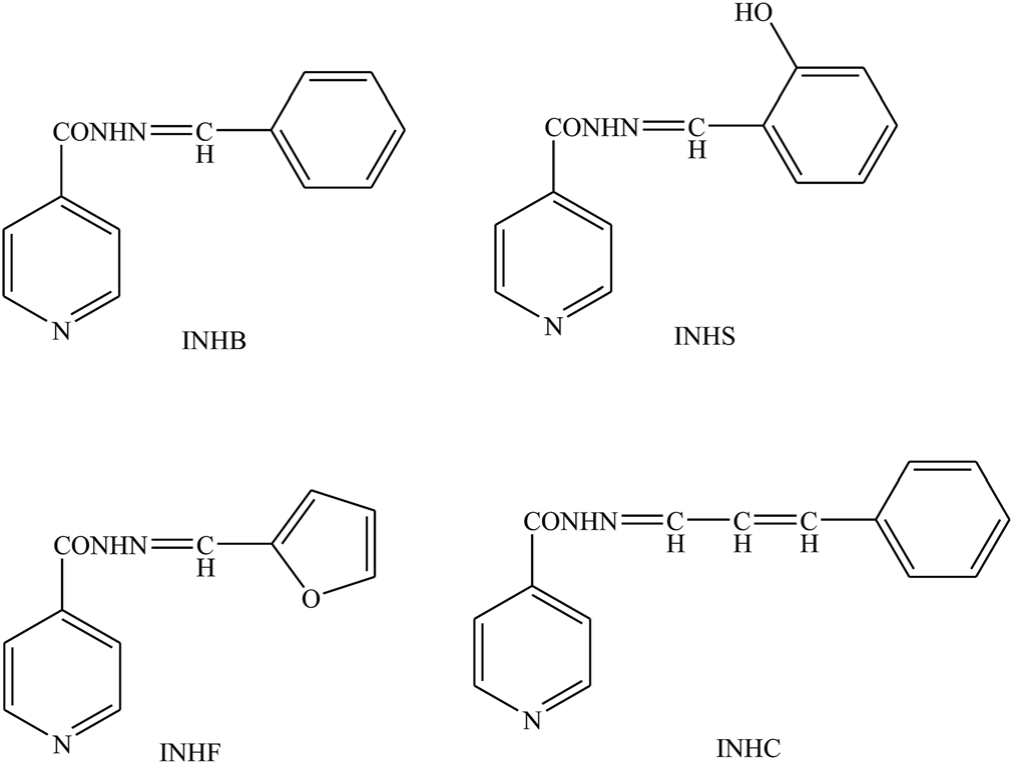
Chemical structure of the tested SBs.

The studied SBs were 2-((1*E*)-2-aza-2-pyrimidine-2-ylvinyl)thiophene, 2-((1*Z*)-1-aza-2-(2-pyridyl)vinyl)pyrimidine (PT), 2-((1*E*)-2-aza-2-(1,3-thiazol-2-yl)vinyl)thiophene, and 2-((1*Z*)-1-aza-2-(2-thienyl)vinyl)benzothiazole (PP) (Fig. 5). Electrochemical results indicated that SBs act essentially as an anodic inhibitor. The difference in inhibitive efficiency of the organic inhibitor mainly depends on the type and nature of heteroaromatic rings present in the chemical molecule. As a result from this work, the difference in inhibition efficiency between PT and PP arising due to the presence of the π electron excess ring (*i.e.* thiophene) in PT instead of the presence of the π electron deficient ring (*i.e.* pyridine) PP as a substituent. In this regard, the presence of the releasing π electron excess ring in PT causes an increase in the electron density of the imine groups (CH=N) in the chemical structure which offers a better protective action of the metal than PP.^83^ In addition, the inhibition effect of SBs (Fig. 6 and 7) on the corrosion of metal in 1 M HCl has been investigated by polarization and electrochemical impedance spectroscopy. The polarization curve results revealed that SBs act as mixed type (cathodic/anodic) inhibitors.^84–86^

**Fig. 8:**
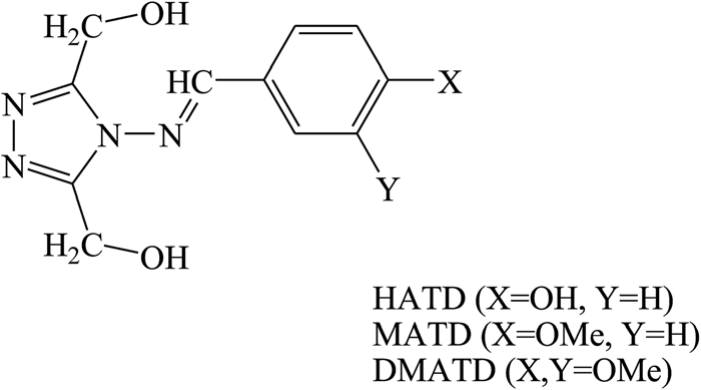
Structure of inhibitor molecule.

Three triazole Schiff bases, namely (4-(4-hydroxybenzylideneamino)-4*H*-1,2,4-triazole-3,5-diyl)dimethanol (HATD), (4-(4-methoxybenzylideneamino)-4*H*-1,2,4-triazole-3,5-diyl)dimethanol (MATD) and (4-(3,4-dimethoxybenzylideneamino)-4*H*-1,2,4-triazole-3,5-diyl)dimethanol (DMATD) (Fig. 8) for metal corrosion in HCl have been examined by electrochemical, spectroscopic and computational studies. The results showed that these synthesized compounds act as effective inhibitors for mild steel in HCl. The inhibition efficiency of these organic compounds increases with their concentration on the metal surface, whereas it decreases with temperature and HCl concentration. The corrosion measurement values indicate that in the presence of inhibitors, the displacement in the value is <85 mV when compared to that of the blank solution, which proposes a mixed type behavior for SBs.^87^

**Scheme 21:**
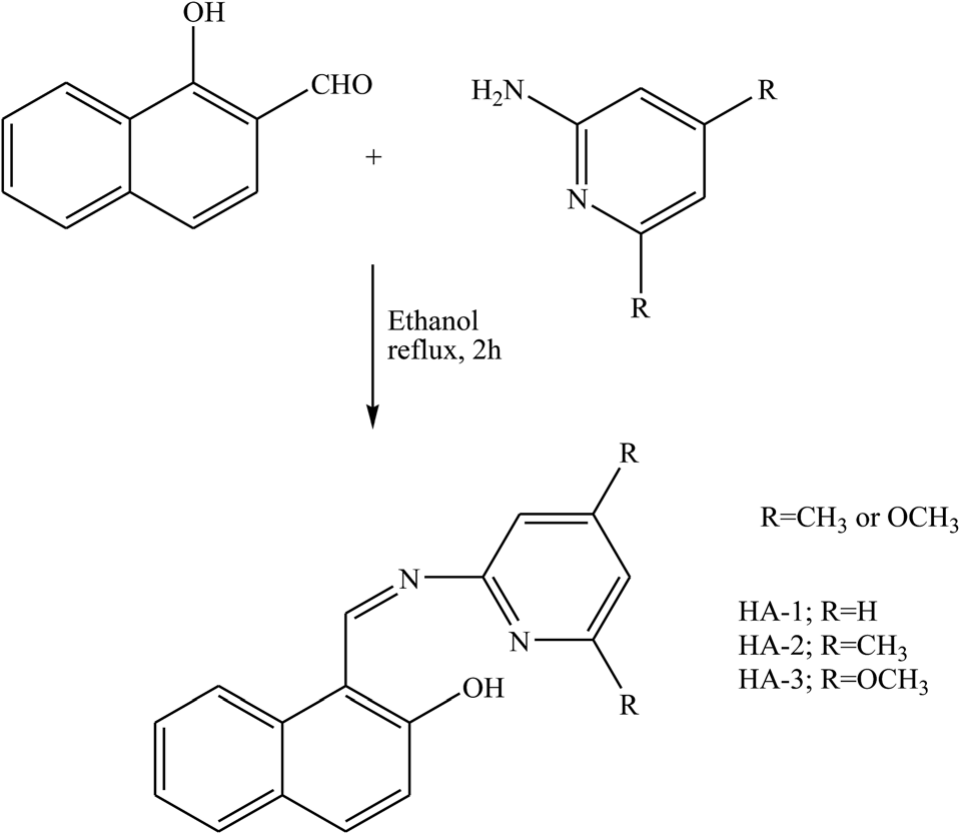
Representation procedures for the synthesis of Schiff base compounds.

El-Lateef *et al.*^88^ have reported synthesis and characterization three SB compounds (Scheme 21) by X-ray, ^13^C-NMR, ^1^H-NMR, mass, UV-vis, FT-IR, spectral data, and elemental analyses. Moreover, the corrosion inhibition of the studied inhibitors towards carbon steel in 15% HCl was examined by using electrochemical measurements, SEM, and EDX. The result in this work confirmed that the formation of the adsorbed layer of SBs provided the protective property against corrosion, resulting in excellent corrosion inhibition. Therefore, the presence of HA-3 as an organic inhibitor results in a comparatively cleaner and smoother surface. This finding elucidates a good physical adsorption and corrosion inhibition potentiality of HA-3 compared to HA-1.

**Scheme 22:**
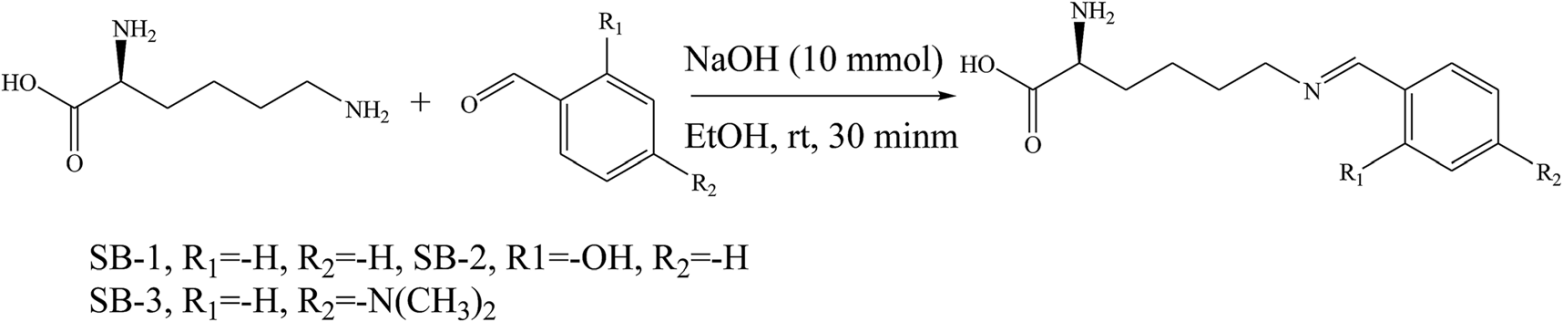
Synthetic route for investigated SBs.

**Scheme 23:**
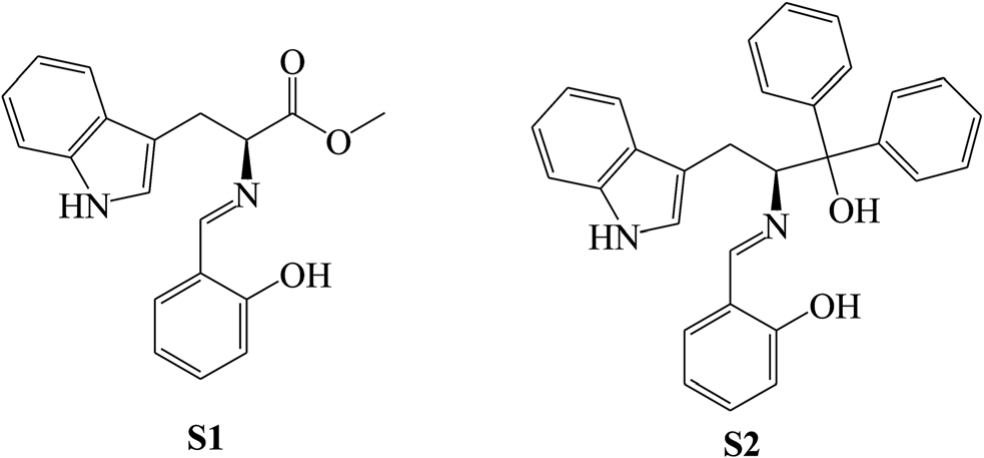
Chemical structure of Schiff base molecules S1 and S2.

SBs, namely 2-amino-6(2-hydroxybenzelideneamino)hexanoic acid (SB-1), 2-amino-6-(4-methoxybenzelideneamino)hexanoic acid (SB-2) and 2-amino-6-(4-dimethylamino)benzyli-deneamino)hexanoic acid (SB-3) (Scheme 22), derived from lysine and three different aldehydes were prepared and evaluated as organic inhibitors for metals in HCl solution using electrochemical methods. The data showed that the inhibition efficiency increases with the increasing concentration of the SBs. Electrochemical measurements revealed that the studied SBs act as cathodic-type inhibitors. It is shown that the addition of SBs retards both cathodic and anodic reactions; however, the cathodic reactions are comparatively more affected than the anodic reactions, suggesting that the investigated SBs are mixed-type inhibitors and act predominantly as cathodic inhibitors.^89^ In addition, some researchers have studied the effect of SBs derived from l-tryptophan, methyl 2-((2-hydroxybenzylidene)amino)-3-(1*H* indol-3-yl)propanoate (S1) and 2-(((1-hydroxy-3-(1*H*-indol-3-yl)-1,1-diphenylpropan-2-yl)imino)methyl)phenol (S2) (Scheme 23), on the corrosion performance of stainless steel was examined. They found that the inhibitors S1 and S2 have good inhibition efficiency on the corrosion of metals in HCl solution. Polarization tests confirmed a mixed mode of inhibition of S1 and S2 with predominant control of the cathodic reaction.^90^

**Scheme 24:**
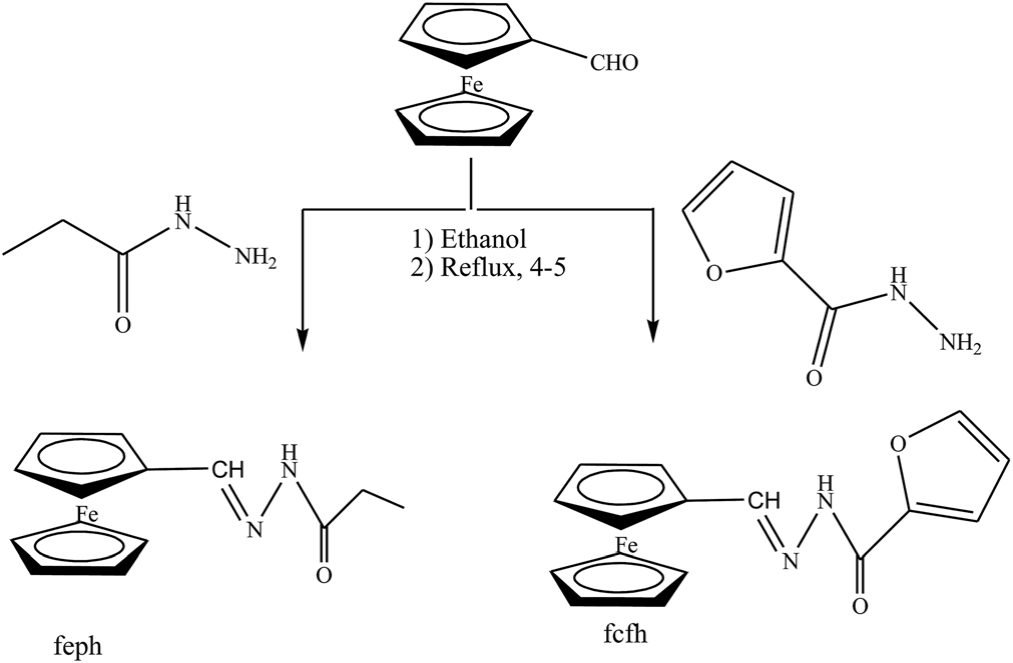
Synthesis of ferrocene carboxaldehyde propanoylhydrazone (fcph) and ferrocene carboxaldehyde furoylhydrazone (fcfh) Schiff bases.

**Scheme 25:**
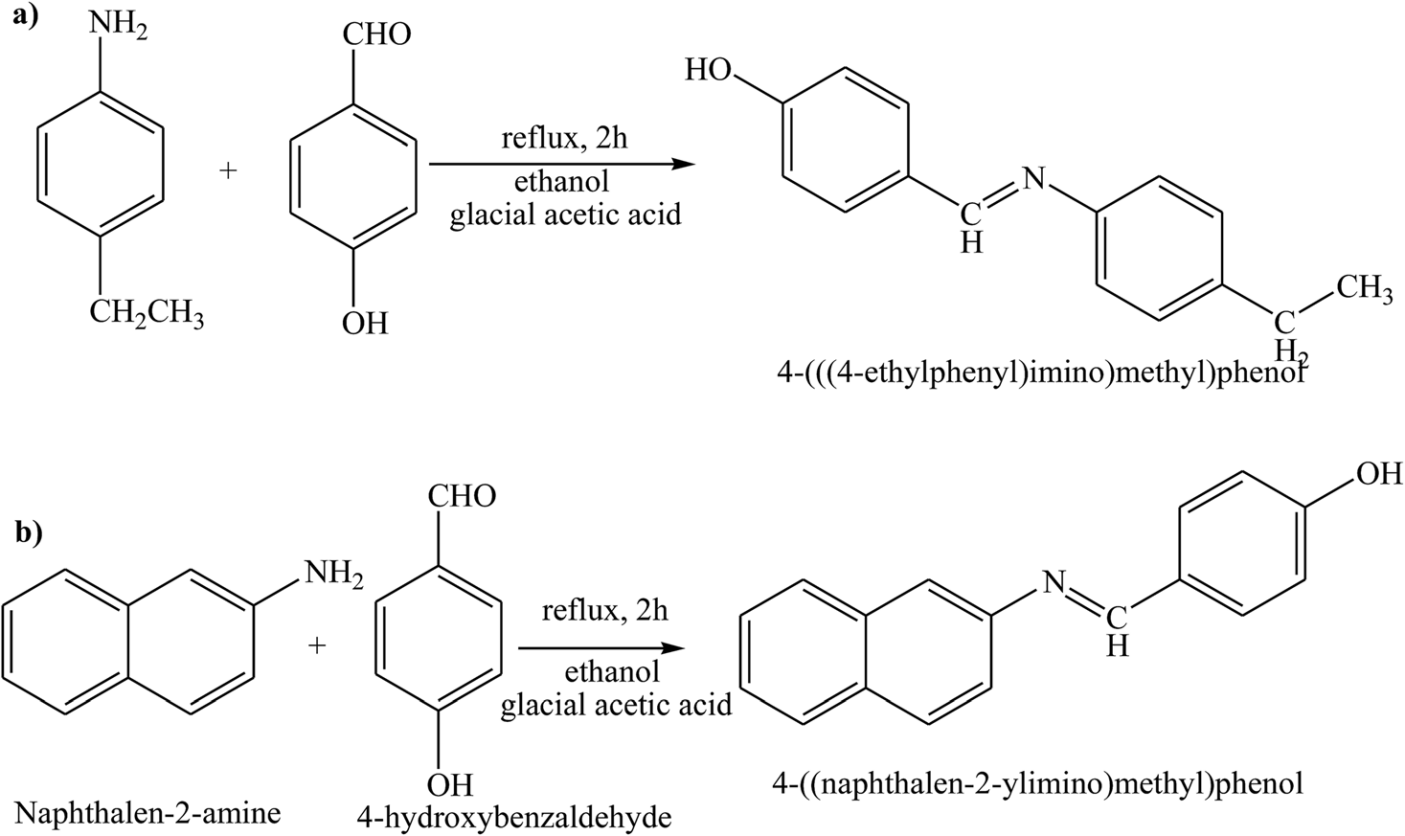
Reaction pathway showing the formation (*E*)-4-((naphthalen-2-ylimino)methyl)phenol Schiff base.

Gupta *et al.* have reported the synthesis and characterization of SBs by elemental analyses, electronic, IR and NMR spectral studies. In this study the structures of the SBs (Scheme 24) were stabilized by intermolecular H-bonding. On the other hand, the electrochemical performance of the SBs exhibits quasi-reversible one-electron redox behaviour. Moreover, the corrosion inhibition studies of SBs have been carried out by using potentiodynamic polarization and electrochemical impedance spectroscopy (EIS) tests. Further, the reduction in anodic and cathodic current densities at a given applied potential shows that these SBs inhibit the reduction of hydrogen ion as well as dissolution of the metal.^91^ On the other hand, 4-(((4-ethylphenyl)imino)methyl)phenol (4 EMP) and (*E*)-4-((naphthalen-2-ylimino)methyl)phenol (4 NMP) (Scheme 25) have been prepared by the reaction of 4-hydroxybenzaldehyde with 4-ethylaniline (for 4 EMP), or naphthalene-2-amine (for 4 NMP). The electrochemical performance of these SBs was investigated on a metal surface in 1 M HCl solution, and was tested using potentiodynamic polarization (PDP) and electrochemical impedance spectroscopy (EIS). The results of the electrochemical procedures showed that the studied molecules imparted high resistance in allowing the flow of electrons across the metal–electrolyte platform and behaved as mixed-type inhibitors, with 4 EMP showing better inhibition properties than 4 NMP.^92^

**Scheme 26:**
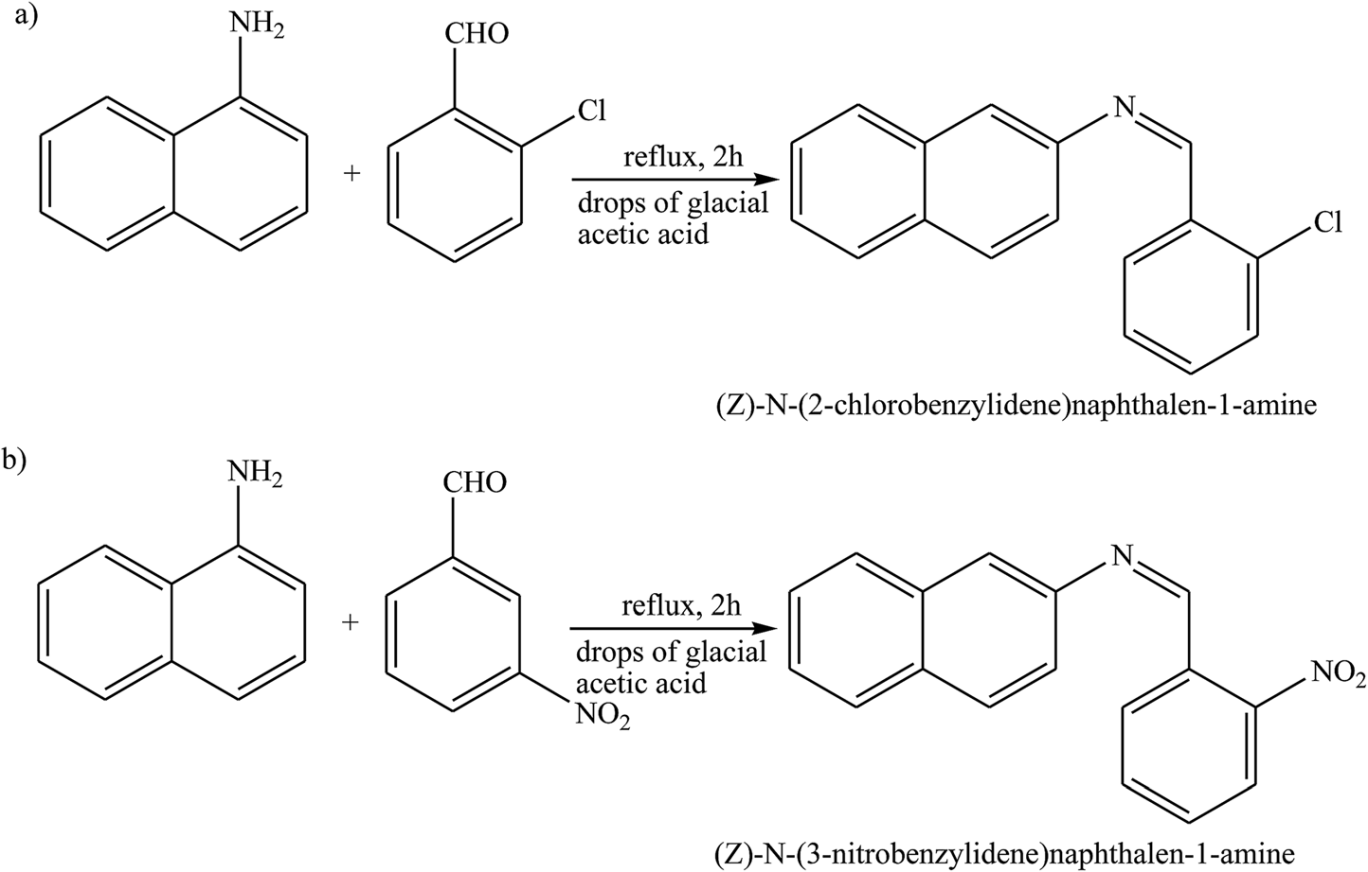
Reaction route for the preparation of (a) (*Z*)-*N*-(2-chlorobenzyllidene)naphthalene-1-amine, and (b) (*Z*)-*N*-(3-nitrobenzyllidene)naphthalene-1-amine.

**Scheme 27:**
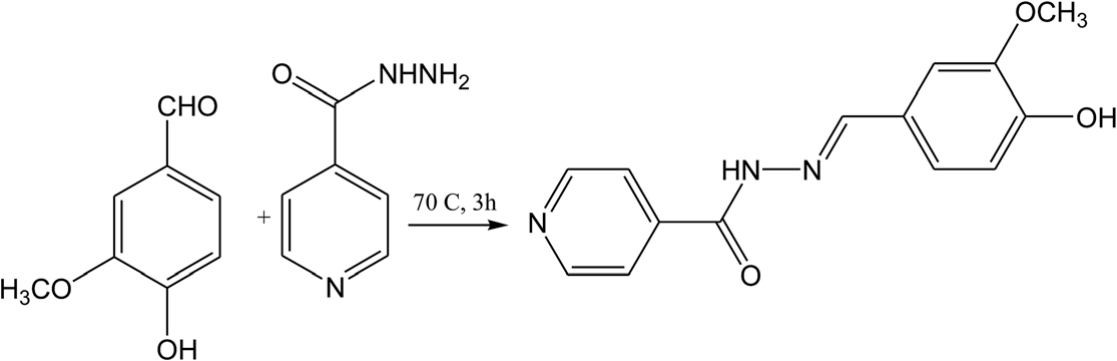
Reaction of vanillin with isoniazid.

**Fig. 9:**
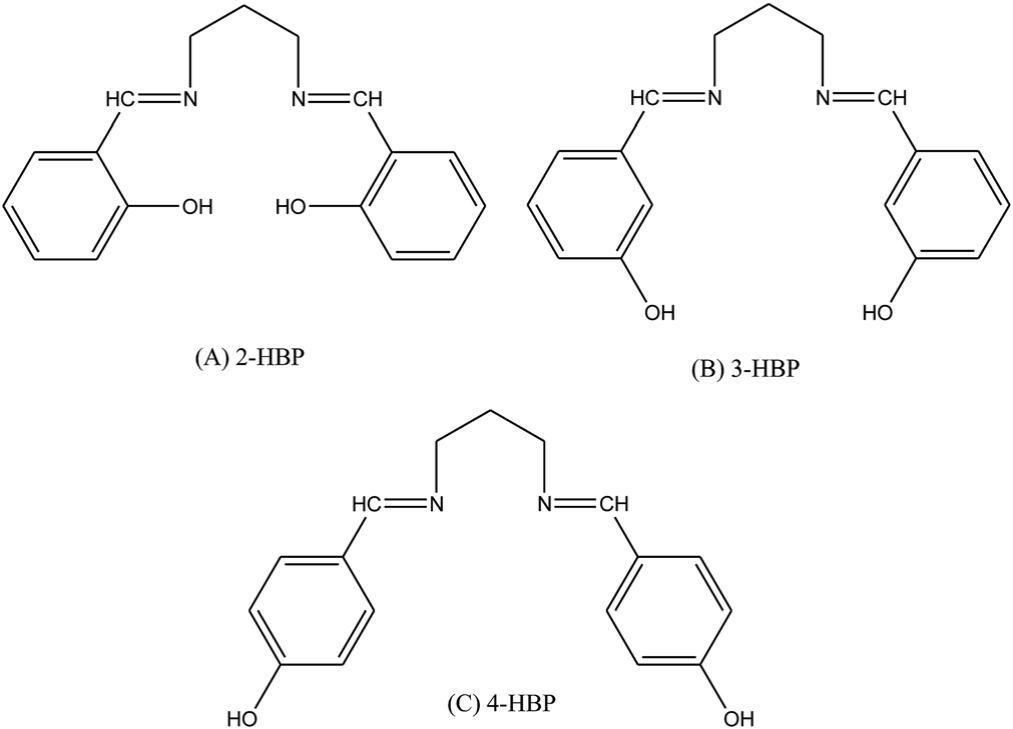
Chemical structures of the Schiff bases.

Schiff base compounds (*Z*)-*N*-(2-chlorobenzylidene)naphthalen-1-amine (CBN) and (*Z*)-*N*-(3-nitrobenzylidene)naphthalen-1-amine (NBN) (Scheme 26) have been prepared by the reaction of 1-naphthylamine and 2-chlorobenzaldehyde or 3-nitrobenzaldehyde respectively. The structures of the compounds were further verified using density functional theory (DFT) which showed the role of the electrons towards corrosion inhibition. Electrochemical measurements using potentiodynamic polarization and electrochemical impedance spectroscopy revealed that both compounds are very good corrosion inhibitors, with CBN showing better properties. Therefore, an increase in the SB concentration with inhibition efficiency is accompanied by a decrease in *I*_corr_.^93–95^ In addition, a Schiff base (*N*-isonicotinamido-3-methoxy-4-hydroxybenzalaldimine, IM) compound (Scheme 27) based on the flavoring (vanillin) and medicine (isoniazid) has been prepared and utilized as a green corrosion inhibitor for copper in aggressive media. Electrochemical techniques are carried out to analyze its inhibition property. However, the current density of the cathodic part changes slightly compared to that of the blank plot. Therefore, the *E*_corr_ value of the Cu electrode shifts to positive after the addition of the inhibitor. These phenomena reveal that the influence of the inhibitor on the anodic metal dissolution is more pronounced than that on the cathodic reduction, and IM can be characterized as an anodic inhibitor.^96,97^ Therefore, the corrosion inhibition and adsorption of *N*,*N*′-bis(*n*-hydroxybenzaldehyde)-1,3-propandiimine (*n*-HBP) (Fig. 9) SBs have been investigated on steel electrode in 1 M HCl by using electrochemical techniques. Therefore, the results propose that the highest inhibition efficiency was obtained for 3-HBP. Polarization curves reveal that all studied inhibitors are of mixed type.^98^

**Fig. 10:**
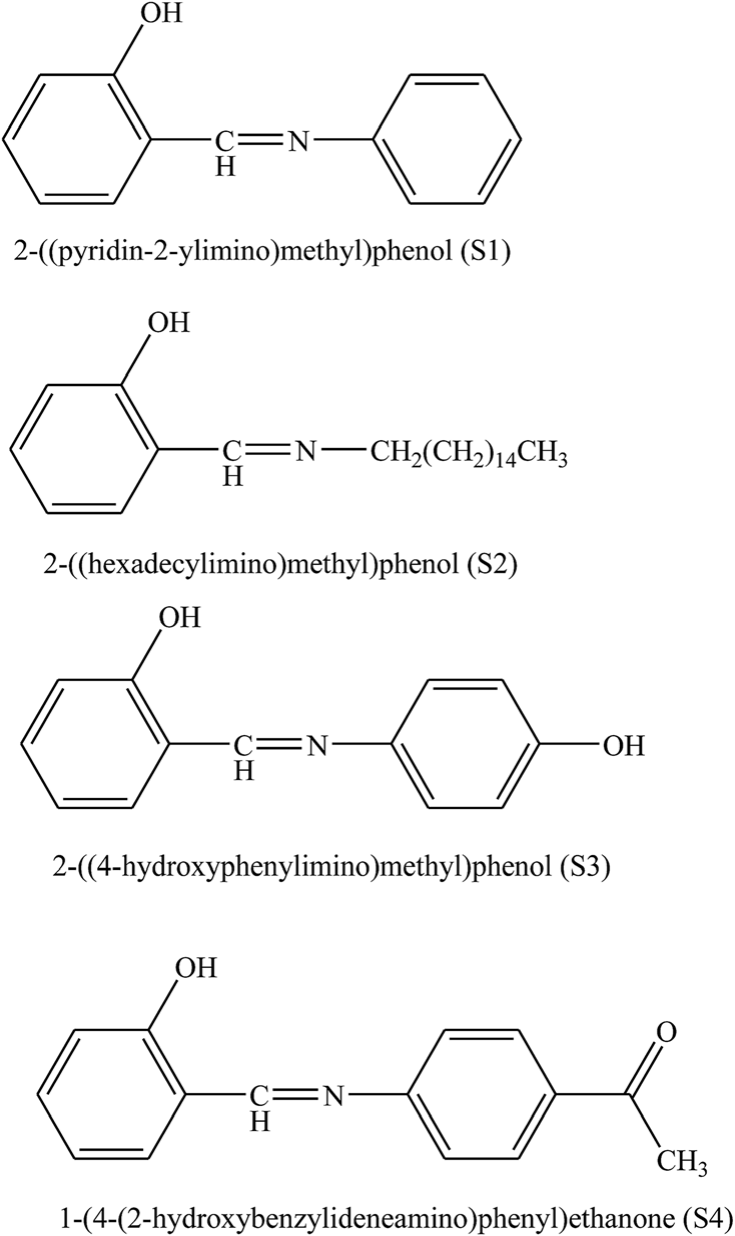
Chemical structures of the synthesized inhibitors.

The corrosion inhibition effect of *o*-aminophenol-*N*-benzylidene (*o*-AmphNB) and *o*-anisidene-*N*-benzylidene (*o*-AnsNB) (Fig. 10) for aluminium in 1 M HCl at different concentrations of the two different SBs has been studied by means of potentiodynamic polarization and electrochemical impedance spectroscopy (EIS). In this work, potentiodynamic polarization parameters exhibited the mixed mode of inhibition with predominance of cathodic inhibition. The impedance results revealed that with a rise in the concentration of the inhibitor, the charge transfer resistance increases while the double layer capacitance decreases.^99^ On the other hand, the corrosion inhibition efficiency of 2-((pyridin-2-ylimino)methyl)phenol (S1), 2-((hexadecylimino)methyl)phenol (S2), 2-((4-hydroxyphenylimino)methyl)phenol (S3), and 1-(4-(2-hydroxybenzylideneamino)phenyl)ethanone (S4) for carbon steel in 1 M HCl has been studied. Polarization and electrochemical impedance spectroscopy experiments showed that SBs are the best used inhibitor. The results of electrochemical impedance and Tafel polarization measurements consistently showed that, all compounds are good inhibitors. Polarization curves indicated that the studied SBs acted as mixed (cathodic/anodic) inhibitors. The differences in the corrosion inhibition efficiency between four studied SBs are correlated with their chemical structures.^100^

**Fig. 11:**
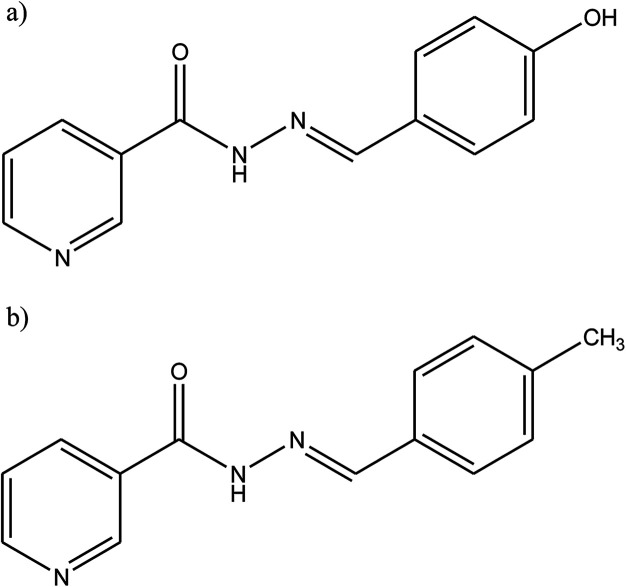
Chemical structure of the synthesized corrosion inhibitors: (a) *N*′-(4-hydroxybezylidene)nicotinic hydrazone (HBNH), and (b) *N*′-(4-methylbezylidene)nicotinic hydrazone (MBNH).

SB derivatives, namely *N*′-(4-hydroxybezylidene)nicotinic hydrazone (HBNH) and *N*′-(4-methylybezylidene)nicotinic hydrazone (MBNH) (Fig. 11), have been studied as organic inhibitors for iron in 1 M HCl using electrochemical methods. The results show good corrosion inhibition efficiency, with the inhibition efficiency of MBNH being higher than that of HBNH. This can be explained through the protonation of the hydroxyl group in 1 M HCl solution decreasing the π-electron density in the aromatic ring of HBNH which causes a weaker physical interaction between the metal surface and HBNH molecules as compared to that in case of MBNH. Moreover, both compounds can be classified as mixed-type inhibitors with a predominant in cathodic effect.^101^

**Fig. 12:**
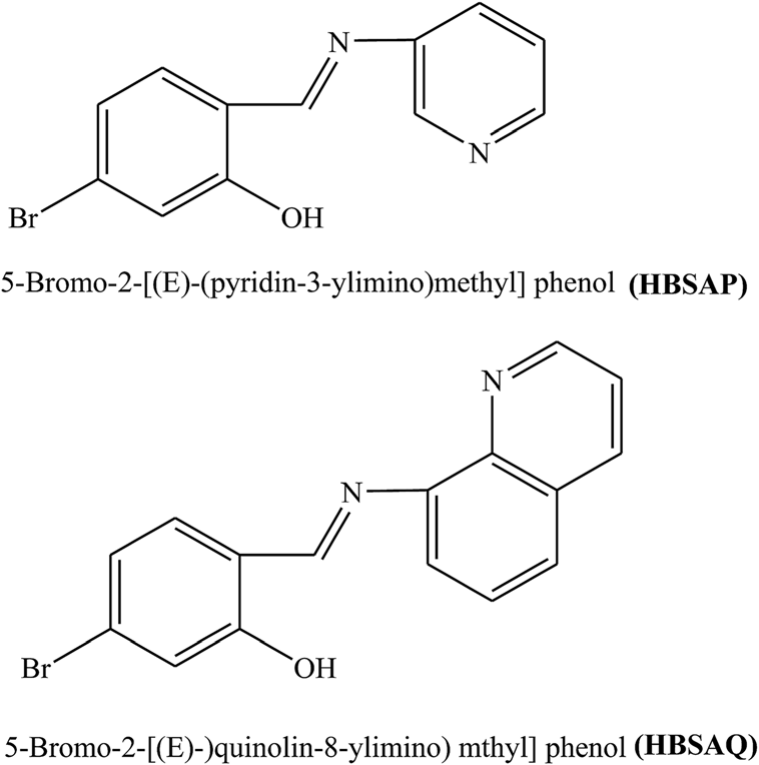
Chemical structures of the synthesized SBs.

Two SBs, 5-bromo-2-[(*E*)-(pyridin-3-ylimino)methyl]phenol (HBSAP) and 5-bromo-2-[(*E*)-(quinolin-8-ylimino)methyl]phenol (HBSAQ) (Fig. 12) have been prepared. The inhibition activity of these SBs for metal in aggressive solution with 0.1 M HCl for both short and long immersion times was investigated using electrochemistry and surface characterization. Potentiodynamic polarization revealed that the organic molecule is more adsorbed on the cathodic sites. Therefore, its corrosion efficiency increases with increasing organic inhibitor concentration.^102^ In addition, the higher values of inhibition efficiencies were possibly due to the presence of the imine group and conjugated and aromatic molecules. As a result, the sequence of inhibition efficiency found from the electrochemical measurements to be as follows: HBSAQ > HBSAP. The higher inhibition efficiency of organic inhibitor HBSAQ may be assigned to the presence of one more aromatic and conjugated ring in the structure of these SBs. Omanovic and Metikos-Hukovic^103^ stated that the stereo size of the adsorbed molecules influences the inhibitory properties of SBs.

**Fig. 13:**
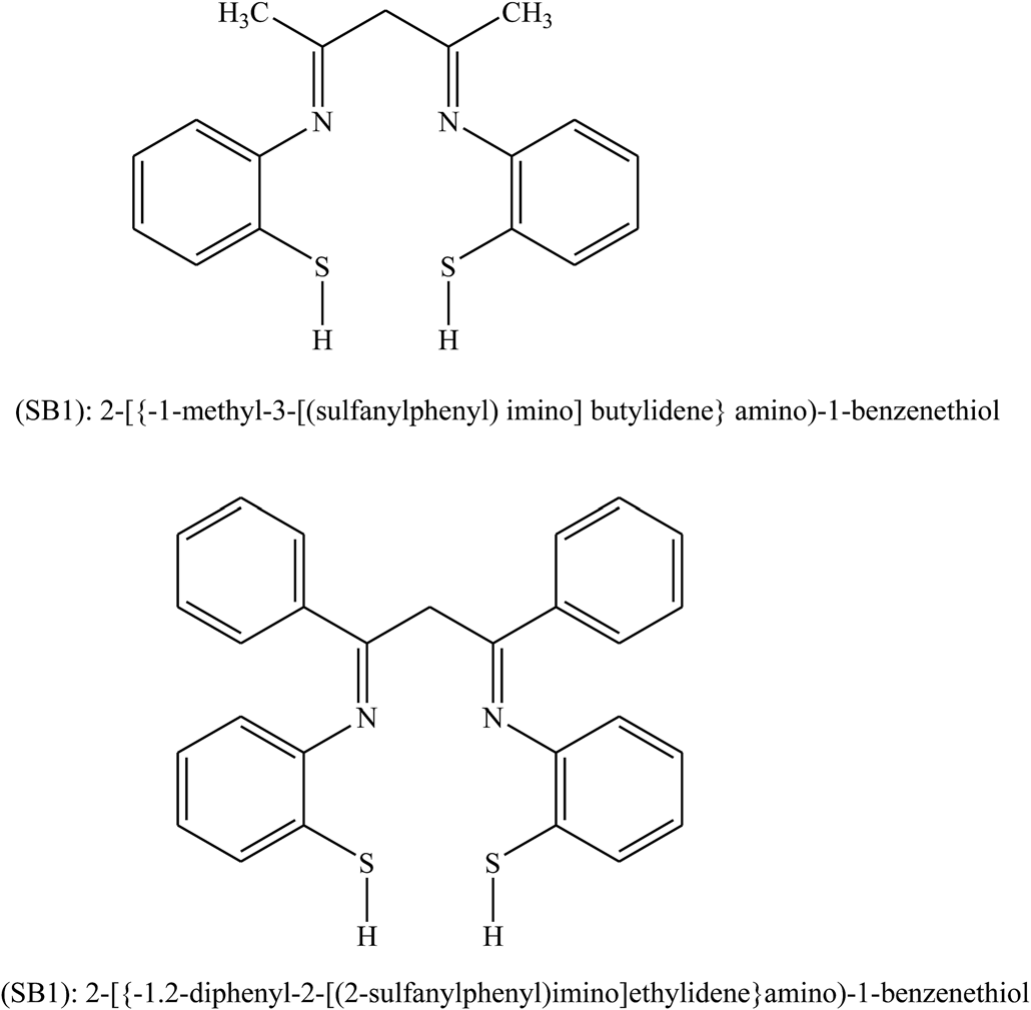
Structure of the investigated SBs.

Behpour *et al.*^104^ have investigated the inhibiting action of SBs, namely SB1: 2-({-1-methyl-3-[(2-sulfanylphenyl)imino]butylidene}amino)-1-benzenethiol and SB2: 2-({-1,2-diphenyl-2-[(2-sulfanylphenyl)imino]ethylidene}amino)-1-benzenethiol (Fig. 13) on the corrosion of metal in HCl. The SBs were prepared and examined as organic inhibitors for the corrosion of Cu metal. Electrochemical measurements consistently identify both compounds as good organic inhibitors. Therefore, impedance spectroscopy (EIS) discovered that the corrosion of Cu in HCl was influenced to some extent by mass transport since the Warburg impedance was shown in some circumstances. Moreover, polarization curves reveal that both of the studied SBs act as mixed type (cathodic/anodic) inhibitors. Differences in inhibition efficiency between SB1 and SB2 are associated with their chemical and stereo structures.

**Fig. 14:**
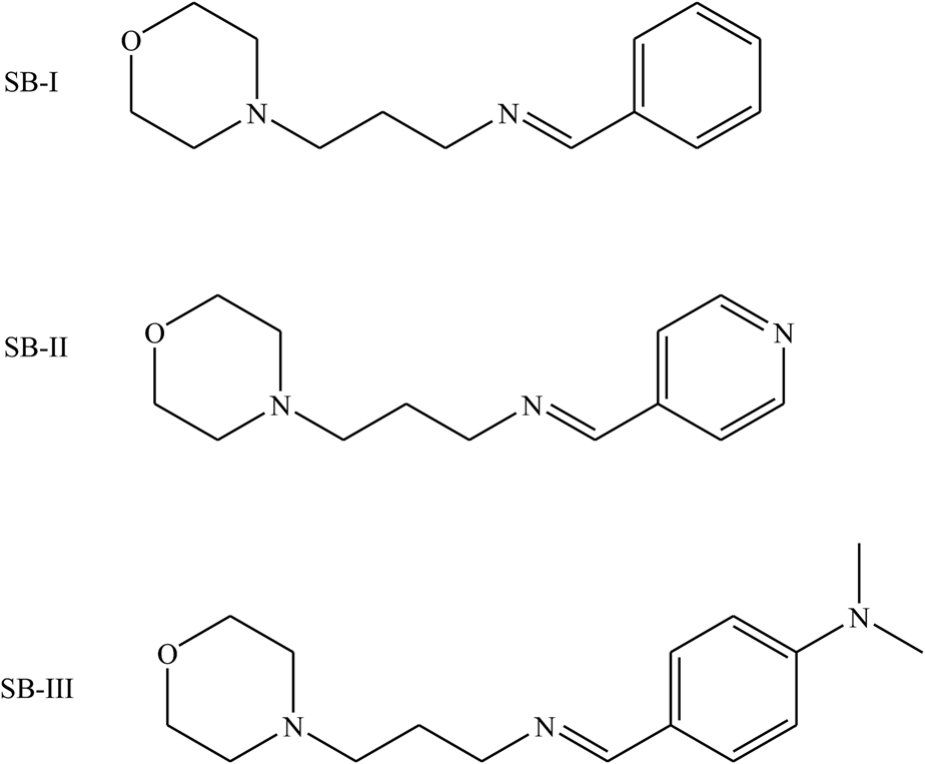
Chemical structure of SBs.

Other works^105^ have reported the inhibition effect of three SBs (SB-I, SB-II and SB-III) (Fig. 14) and their synergistic effect with KI on the corrosion of iron in acidic solution by electrochemical measurements such as potentiodynamic polarization and electrochemical impedance spectroscopy. Scanning electron microscopy (SEM) was used to characterize the steel surface. The inhibition efficiency increases with the concentration of the Schiff bases and increases further with the presence of KI. The thermodynamic parameters *K*_ads_ and DG_ads_ are calculated and discussed. The probable inhibitive mechanism is proposed from the viewpoint of adsorption theory. According to the results in this work, the *I*_corr_ value decreases substantially, leading to higher inhibition efficiency of the KI/SB (SB-I, SB-II and SB-III) mixtures, up to 98.6%, 98.9% and 99.4%, compared to 57.6%, 84.7% and 89.5% obtained for 0.0005 M of SB-I, SB-II and SB-III alone, respectively. This reveals a synergistic effect among SBs and KI.^106,107^

**Fig. 15:**
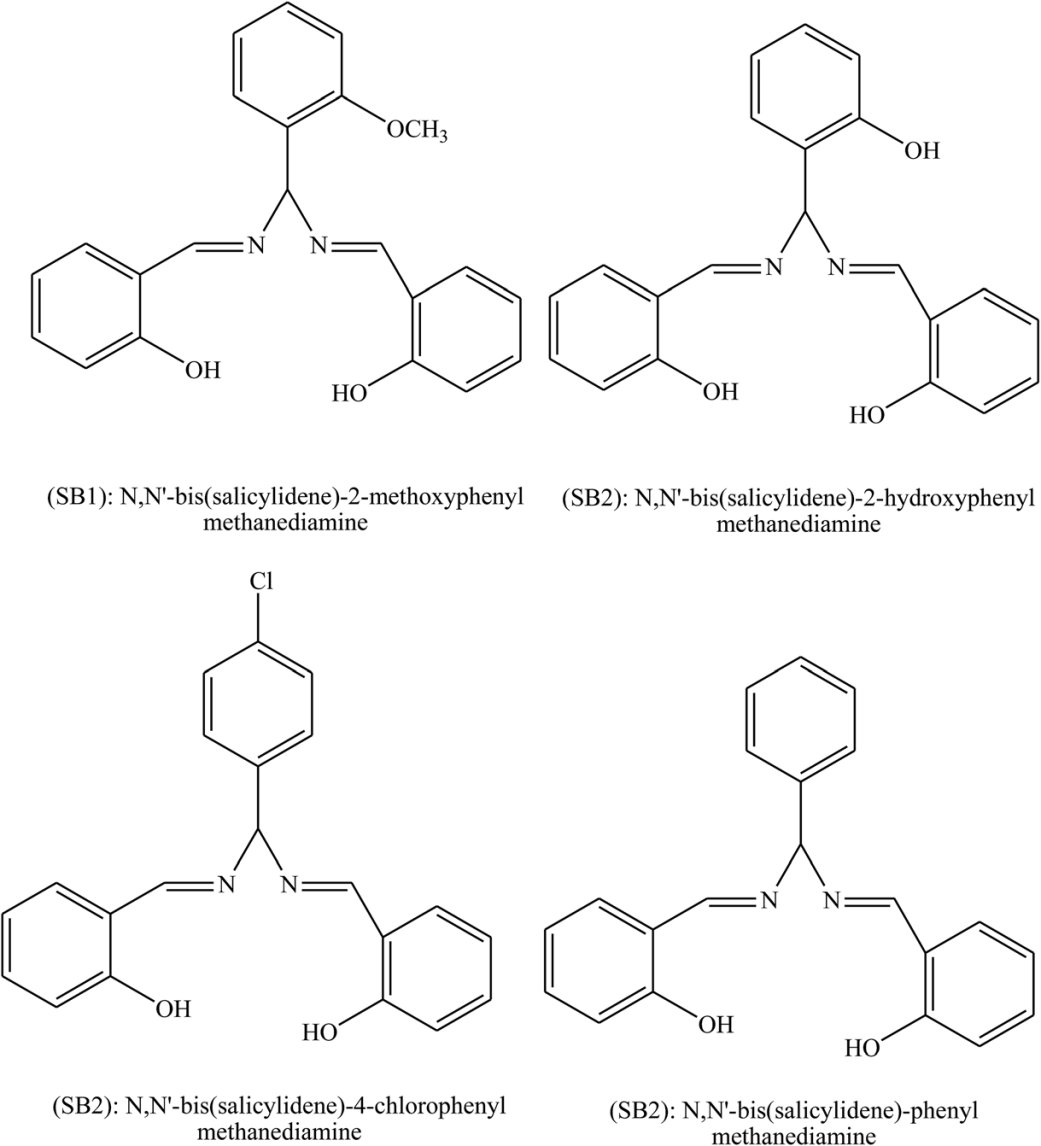
Names and structures of the investigated SBs.

The inhibition effect of SBs (Fig. 15) on the corrosion of iron in HCl has been studied by polarization, electrochemical impedance spectroscopy (EIS) and weight loss measurements. The results of this work show that all studied SBs act as effective organic inhibitors. The corrosion inhibition increases when the concentration of the SBs increases. This study reveals that by increasing the SB concentration, *I*_corr_ was decreased, *E*_corr_ was shifted slightly to more positive values, and the inhibition efficiency IEP (%) increased.^108^

**Fig. 16:**
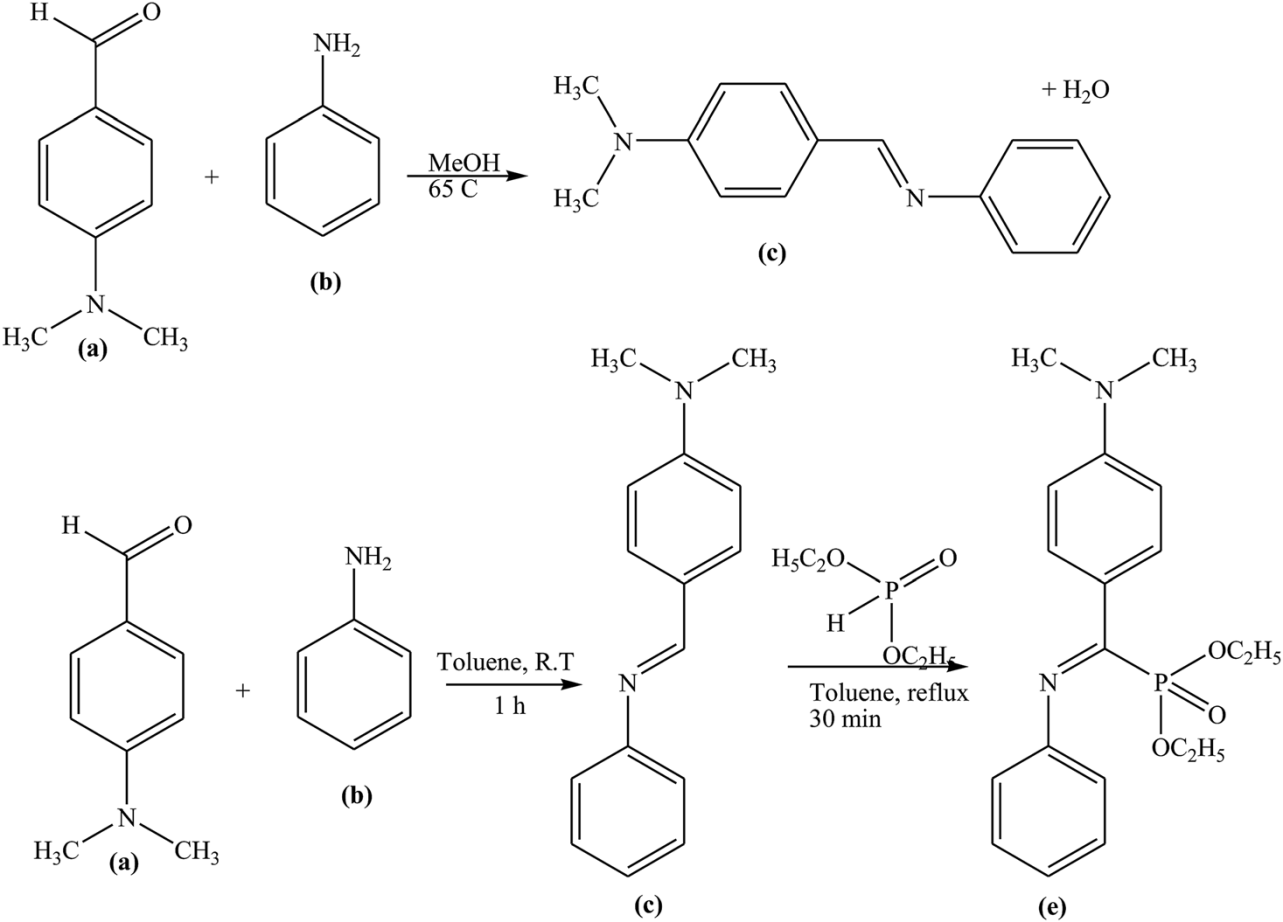
Synthesis route of the studied compounds: (a) 4-(dimethylamino)benzaldehyde, (b) aniline, (c) (*E*)-*N*,*N*-dimethyl-4-((phenylimino)methyl)aniline (E-NDPIMA), (d) diethylphosphite, and (e) diethyl ((4-(dimethylamino)phenyl)(phenylamino)methyl)phosphonate (α-APD).

α-Aminophosphonate (α-APD) and SB (E-NDPIMA) derivatives (Fig. 16) have been synthesized and their structures were proved by IR, UV-vis, ^1^H, ^13^C and ^31^P NMR spectroscopy. Their inhibitive capacities on the corrosion of carbon steel in H_2_SO_4_ solution were ascertained by Tafel polarization and electrochemical impedance spectroscopy (EIS). Experimental results clarified that the prepared compounds are effective inhibitors and the adsorption of organic molecules on the Fe surface obeys the Langmuir adsorption isotherm.^109^

#### 2.2.2 Aluminium

**Fig. 17:**
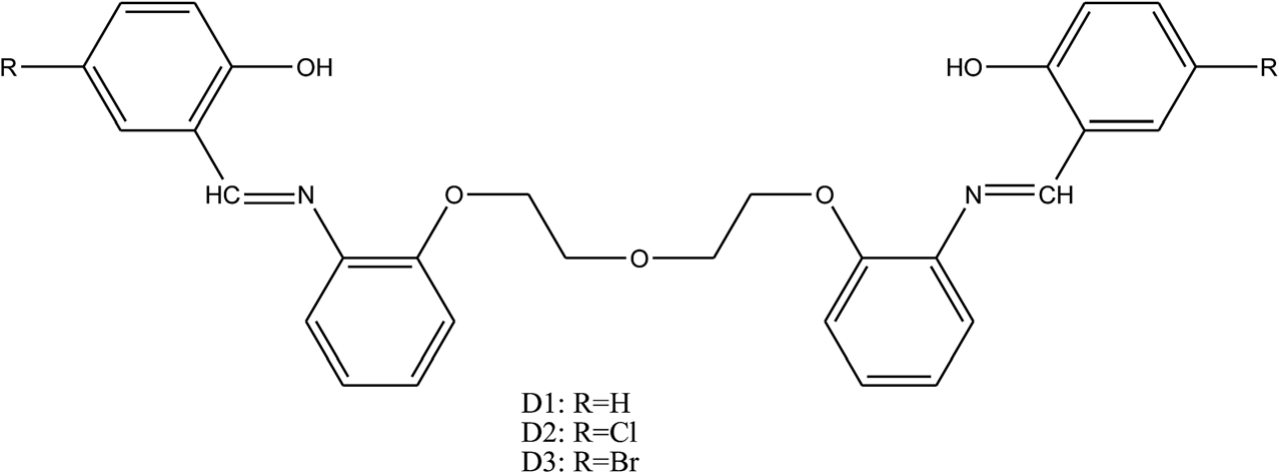
General structure of the investigated SBs.

Safak *et al.*^110^ have reported the synthesis of SBs named 1,5-bis[2-(2-hydroxybenzylideneamino)phenoxy]-3-oxopentane (D1), 1,5-bis[2-(5-chloro-2-hydroxybenzylideneamino)phenoxy]-3-oxopentane (D2) and 1,5-bis[2-(5-bromo-2-hydroxybenzylideneamino)phenoxy]-3-oxopentane (D3) (Fig. 17) and their inhibitive capabilities on the corrosion of aluminium in acidic solution. Electrochemical measurement curves demonstrated that the studied SBs were a cathodic inhibitor and the effectiveness of these inhibitors decreased in the order of D3 > D2 > D1. It means that Br-substituted SB has a more protective ability than the Cl-substituted one, and Cl-substituted SB is more protective compared to its unsubstituted derivative. Similar results were documented by previous work^111^ where bromine including a phenolic SB is a more effective inhibitor than its Cl-substituted and unsubstituted derivatives.

**Fig. 18:**
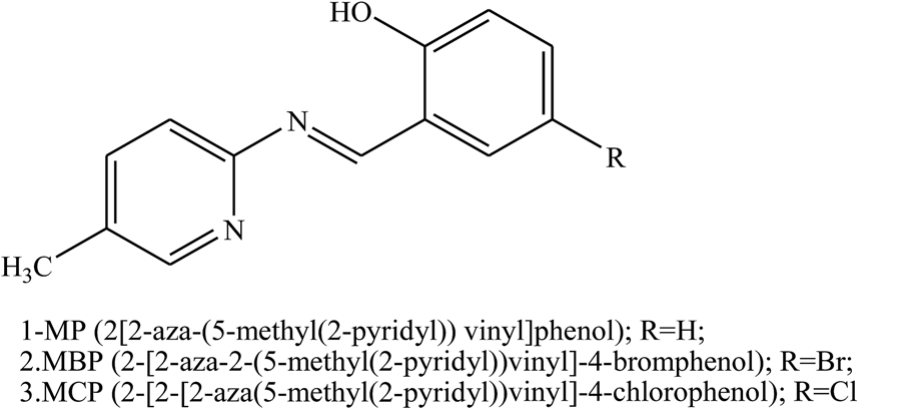
Schiff bases.

Other works investigated the effect of Schiff bases containing aza groups such as 2-[2-aza-2-(5-methyl(2-pyridyl))vinyl]phenol, 2-[2-aza-2-(5-methyl(2-pyridyl))vinyl]-4-bromophenol, 2-[2-aza-2-(5-methyl(2-pyridyl))vinyl]-4-chlorophenol (Fig. 18) on the corrosion behaviour of aluminum in 0.1 M HCl, studied using electrochemical methods. Polarisation curves show that all of the investigated SBs acted as mixed-type inhibitors. Electrochemical measurements show that inhibition efficiencies increase with the rise in organic inhibitor concentration. These results reveal that the inhibitive actions of SBs were due to physical adsorption on the aluminium surface. As a result, the variation in inhibition efficiency values depends on the type of functional groups substituted on the aromatic ring. Therefore, it was found that the presence of Br and Cl atoms in the chemical structure of the investigated SBs facilitate the adsorption of the molecule on the aluminium surface. On the other hand, the adsorption of the investigated SBs depends on the charge density of the adsorption centers and dipole moments.^112^

**Fig. 19:**
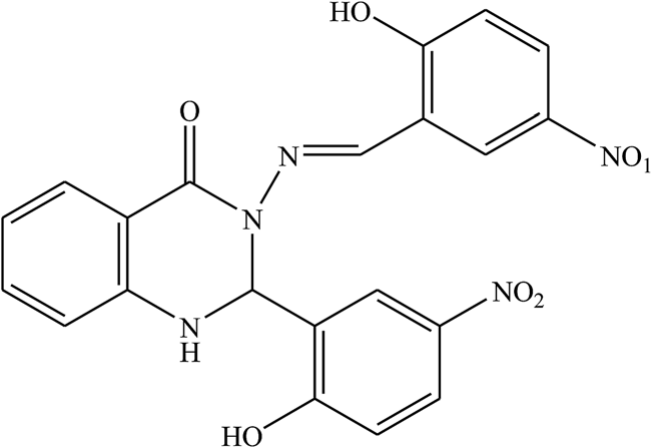
Structure of the investigated SBs: 3-(5-nitro-2-hydroxybenzylideneamino)-2-(5-nitro-2-hydroxyphenyl)-2,3-dihydroquinazoline-4(1*H*)-one (NNDQ).

The inhibitory effect of two SBs, and 3-(5-nitro-2-hydroxylbenzylideneamino)-2-(5-nitro-2-hydroxyphenyl)-2,3-dihydroquinazoline-4(1*H*)-one (NNDQ) (Fig. 19), on the corrosion of metal surface in 1 M HCl acid were investigated using potentiodynamic polarization and electrochemical impedance spectroscopy measurements. The investigation results reveal that the SB compounds with an average efficiency of 92% at an additive concentration of 1.0 mM have fairly effective inhibiting properties for Al metal in HCl, and they show mixed-type inhibitor character. This phenomenon revealed that the SBs may suppress both anodic metal dissolution and the cathodic reaction to produce hydrogen gas with the increase in the SB concentration. The occurrences of CH=N, the phenolic group (–OH), and benzene rings are responsible for their greater activity and inhibition efficiency. The analysis of the results in this work revealed that the presence of weak electron-donating groups, *i.e.* methoxy (–OCH_3_), and the strong deactivating group, the nitro group (–NO_2_), are responsible for the strong interaction of the former inhibitor to the latter one.^113^

**Fig. 20:**
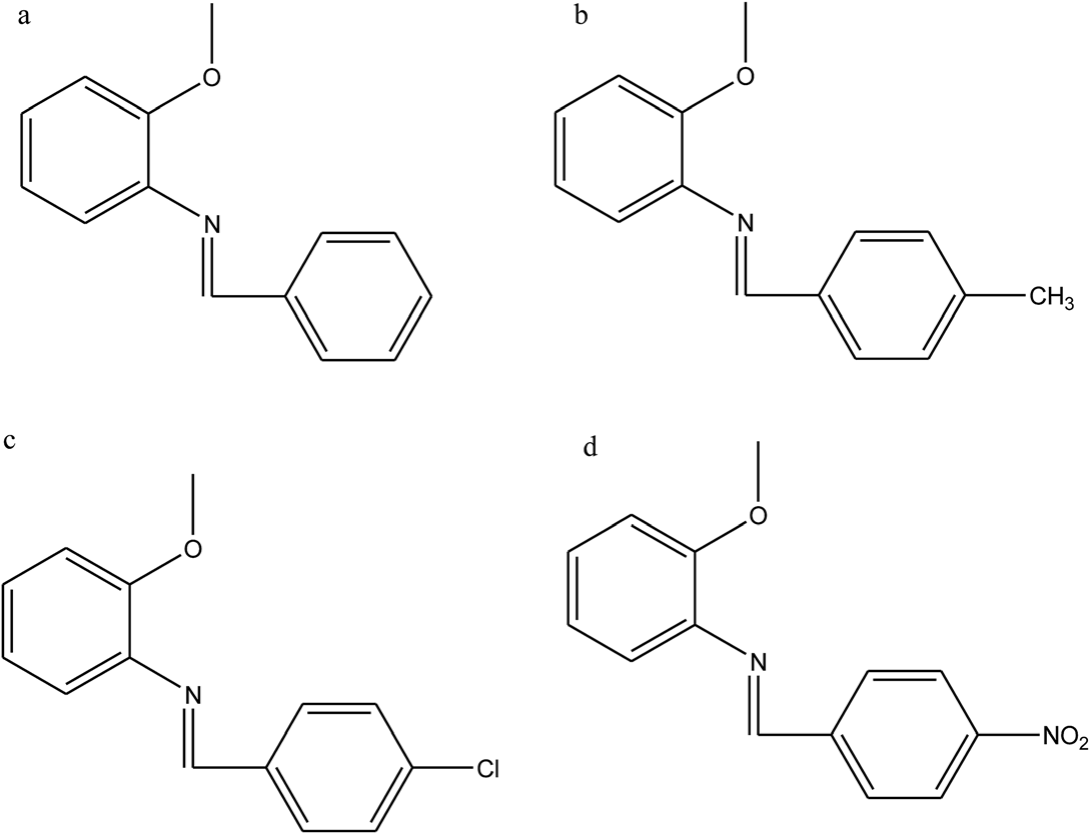
Structure of the studied SBs.

Ashassi-Sorkhabi *et al.*^114^ have reported the study of Schiff bases benzylidene-(2-methoxy-phenyl)-amine (a), (2-methoxy-phenyl)-(4-methyl-benzylidene)-amine (b), (4-chloro-benzylidene)-(2-methoxy-phenyl)-amine (c), and (4-nitro-benzylidene)-(2-methoxy-phenyl)-amine (d) (Fig. 20) on the corrosion of aluminum in 1 M HCl, by polarization and electrochemical impedance spectroscopy (EIS). The results reveal that the inhibition efficiency increases with a decrease in temperature and an increase in the concentration of the Schiff base. The polarization curves show that the SBs used are mixed-type inhibitors. As a result, the performance of the organic compounds depends strongly on the type of functional groups substituted on the conjugated benzene ring.^115,116^

## 3. Conclusion

Imine compounds are considered as a very significant category of organic compounds because of their capability to form complexes with metal ions and because of their pharmacological properties. Imine compounds have been revealed to be promising leads for the design of more competent antimicrobial agents. According to the results of researches on the corrosion protection of metals, various chemical structures of Schiff bases are used for protecting metals from aggressive environments. Therefore, the review of organic inhibitors used for different metals under various conditions makes it possible to choose a competent and cost-effective organic inhibitor for a specific environment. However, acidic or Cl-containing environments are the most aggressive surroundings for metals. The corrosion inhibition performance of organic inhibitors in these aggressive surroundings is different. Some of the chemical structures protect the surface of the metal through the formation of a protective oxide layer and some of them decrease corrosion attack through physical and chemical adsorption and formation of a complex layer. According to the results from previous works, an increase in the concentration of organic inhibitor in the bulk of the solution and an increase in the immersion time result in enhancing the corrosion inhibition behaviour. Moreover, an increase in the temperature of the adsorption process leads to a decrease in the corrosion inhibition efficiency. The other hand of this work was to review the molecular structure of various imine inhibitors, which are used for the protection of different metals. The molecular structure plays a primary role in its corrosion protection. The presence of heteroatoms such as nitrogen, sulfur and oxygen with free electron pairs, alkyl substituents and aromatic rings in the structure of the organic inhibitors play a significant role in their protective behaviors. In this study, therefore, a review of the corrosion protection of metals in common environments such as acidic media or aggressive solution for metals has been given.

## Conflicts of interest

The authors declare no competing financial interest.

## Supplementary Material
